# Tumor Microenvironment Hijacks and Accelerates a Physiological Myeloid Senescence Signature Associated with Pan-Cancer Immunosuppression and Prognostic Stratification

**DOI:** 10.3390/ijms27135688

**Published:** 2026-06-24

**Authors:** Han Jiang, Yakun Zhang, Caiyu Zhang, Tengyue Li, Qianyi Lu, Jiajun Zhou, Jiayi Yang, Jialu Zhang, Yue Gao, Shangwei Ning

**Affiliations:** College of Bioinformatics Science and Technology, Harbin Medical University, Harbin 150081, China; 202301212@hrbmu.edu.cn (H.J.); zyk15776452861@163.com (Y.Z.); 2020020493@hrbmu.edu.cn (C.Z.); travilucas@163.com (T.L.); luqianyi2018@126.com (Q.L.); zzhoujiajun1122@163.com (J.Z.); yjybio@163.com (J.Y.); zjl13080390729@163.com (J.Z.)

**Keywords:** pan-cancer, myeloid, physiological aging, tumor immunosenescence, DAB2^+^ macrophages

## Abstract

Immunosenescence is a critical driver of tumor initiation and progression. In this study, we systematically characterized immune cell senescence by integrating transcriptomic profiles from 17 physiologically aged tissues with pan-cancer single-cell datasets, encompassing 206 samples across nine cancer types. Cross-tissue comparison of senescence-associated alterations, integrated with spatial transcriptomics, revealed that malignant cells triggered senescence in the core myeloid subpopulation designated Mac_DAB2 via a conserved MIF-CD74 signaling axis. By integrating shared myeloid differentiation programs across normal tissues and the tumor microenvironment (TME) with their transcriptional regulatory networks, we defined a myeloid senescence-associated gene (MSAG) signature. This signature successfully distinguishes a senescence-associated, immunosuppressive subtype linked to poor prognosis in pan-cancer cohorts. Finally, we established the MSAG.SIG prognostic model using an ensemble framework of 117 machine learning algorithms, which demonstrated robust and consistent predictive performance across multiple independent cohorts. Overall, this study elucidates the mechanisms underlying TME-driven myeloid senescence, establishes MSAG as a conceptual framework for characterizing myeloid immunosenescence, and provides a clinically relevant pan-cancer prognostic tool with translational potential.

## 1. Introduction

Aging has now been formally recognized as a fundamental hallmark of cancer [1]. Epidemiological evidence consistently shows that cancer incidence rises sharply with age, highlighting the critical role of aging-associated biological changes in promoting tumor initiation and progression [2,3,4,5,6]. With advancing age, declining autophagy and clearance mechanisms impair the removal of damage-associated molecular patterns (DAMPs), amplifying chronic immune stimulation and generating a persistent sterile inflammatory drive [7]. However, despite the well-established association between aging and cancer, the precise immune-mediated mechanisms through which physiological aging predisposes tissues to tumor development remain insufficiently defined.

Immunosenescence, defined as a global decline in the structural and functional integrity of the immune system, is characterized by impaired immune cell effector functions and the persistent accumulation of senescent cells within tissues [8]. As Franceschi articulated in the concept of the ‘immunobiography’, immunosenescence does not occur abruptly but arises from the lifelong accumulation of immune stimulation, infection exposure, and tissue damage [9]. During this process, pro-inflammatory signals released by senescent immune cells promote chronic inflammation, which in turn accelerates immune exhaustion, ultimately establishing a self-reinforcing vicious cycle between innate and adaptive immunity [8]. Nevertheless, current studies predominantly focus on adaptive immune components, whereas the contribution of innate immune remodeling, especially within the myeloid lineage, to tumor-associated immunosenescence remains incompletely characterized.

Macrophages, as the most abundant and functionally versatile immune components of the tumor microenvironment (TME), are deeply implicated in various facets of tumor evolution and immune surveillance [10,11]. This pivotal role has propelled the senescence-associated transformation of macrophages to become a burgeoning frontier for deciphering tumor immune evasion [12,13]. Mechanistically, macrophage senescence is primarily induced by a multitude of extrinsic stressors, encompassing senescence-associated secretory phenotype (SASP) signaling [14], pathogen infections [15,16], ionizing radiation [17], hypoxia [18], metabolic stresses such as hyperglycemia [19], and the tumor-associated genetic milieu [20], which collectively trigger cell cycle arrest, functional decline, and the manifestation of senescent phenotypes. This transition results in profound functional impairment: senescence significantly disrupts the intrinsic transcriptional regulatory homeostasis of macrophages, thereby attenuating their phagocytic capacity, autophagic efficiency, and metabolic signaling, while compromising normal inflammatory responses [21,22,23,24]. However, these observations are largely derived from isolated contexts, and a comprehensive pan-cancer framework integrating physiological aging, myeloid lineage evolution, and tumor-associated senescence at single-cell resolution has been lacking. Moreover, the absence of conserved molecular signatures has limited translational application.

To bridge this gap, we first established that physiological aging is tightly coupled with immune microenvironment remodeling, culminating in a myeloid-enriched phenotype. Utilizing single-cell transcriptomics, we characterized a myeloid-centered immunosenescent landscape and elucidated the underlying mechanisms tethering senescence to tumor progression. We further identified a conserved myeloid senescence-associated gene signature (MSAG) from lineage trajectories, bridging physiological aging with tumor immune remodeling. Together, these findings provide a pan-cancer framework for myeloid senescence with prognostic and therapeutic relevance.

## 2. Results

### 2.1. Immune Senescence Exhibits a Conserved Myeloid Bias Across Multiple Tissues and Pan-Cancer Contexts

**Figure 1:**
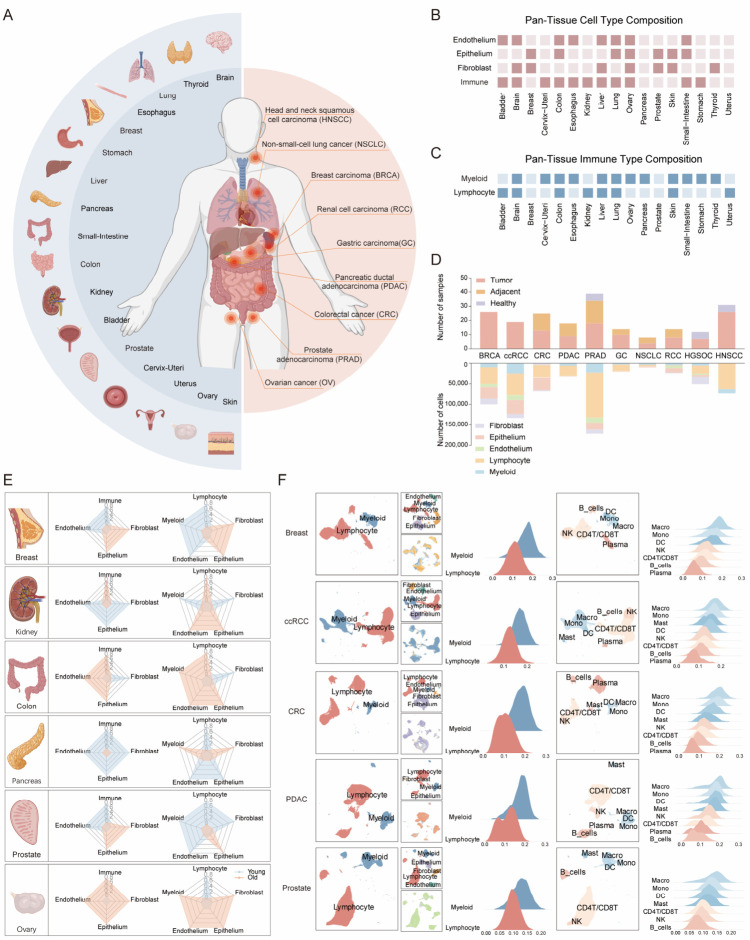
A shared myeloid-dominant immune architecture underlies physiological aging and tumor immunosenescence: (**A**) Overview of tissue types included in the GTEx dataset and cancer types covered by the single-cell transcriptomic datasets. (**B**,**C**) Pan-tissue enrichment frequencies of major cell categories and immune lineages. Heatmaps illustrating cross-tissue distributions derived from binary matrices of ssGSEA scores (1 for significant enrichment in the elderly group, 0 otherwise). (**D**) Summary of single-cell datasets, including the number of samples and corresponding cell counts. (**E**) Radar plots comparing ssGSEA-based cell infiltration scores between elderly and young groups across tissues. (**F**) Pan-cancer immune senescence landscape. UMAPs display cell type annotations and inter-sample batch effects across single-cell datasets. Ridge plots show SAG scores for myeloid and lymphoid cells and their subpopulations. (**A**,**E**) were created in BioRender. Jiang, H. (2026) https://BioRender.com/u5b5d8u, accessed on 15 June 2026.

Normal tissue samples corresponding to 17 common cancer types were collected from the genotype-tissue expression (GTEx) portal (Figure 1A) to investigate physiological immunosenescence. By comparing the enrichment frequency of cell type-specific features between young (20–39 years) and aged (40–79 years) individuals, we found a marked age-associated increase in the enrichment of immune-related cell types (Figure 1B,E and Figure S1A). Further stratification of immune cells into myeloid and lymphoid lineages revealed that aging is associated with the establishment of a myeloid-enriched immune microenvironment (Figure 1C,E and Figure S1A). These findings indicate that physiological immune aging is not characterized by a generalized enhancement of immune components, but instead displays a clear lineage-specific bias.

Given the myeloid-dominated immune infiltration pattern observed in normal tissues, we next extended our analysis to tumor tissues. Using single-cell transcriptomic data from multiple cancer types (Figure 1A), together with candidate senescence-associated genes (SAGs) collected from nine databases and relevant literature sources, we evaluated tumor-associated immunosenescence. Candidate genes supported by at least two independent references were retained, resulting in a curated senescence gene set comprising 769 genes (Figure S1B and Supplementary Table S4). All single-cell datasets were processed under a unified quality control and batch correction pipeline. Cell types were annotated based on canonical markers and classified into five major categories: Myeloid, Lymphocyte, Fibroblast, Endothelium, and Epithelium (Figure S1C). At single-cell resolution, myeloid-dominated immune senescence patterns were highly consistent across cancer types and became even more pronounced at the subpopulation level. Parallel comparisons of major immune subclusters revealed that myeloid-related subsets consistently exhibited significantly higher senescence signals than lymphoid subsets (Figure 1F and Figure S1D,E).

Across both age-related evolution in normal tissues and the complex pathological landscapes of pan-cancer settings, the immune system displays a highly conserved myeloid-biased trajectory. This cross-contextual consistency establishes myeloid cells as a central axis of immune senescence and provides a critical entry point for dissecting the evolutionary logic and driving mechanisms of tumor-associated immune aging.

### 2.2. Tumor Microenvironment Induces and Amplifies Myeloid Senescence Through Directional Signaling Interactions

**Figure 2:**
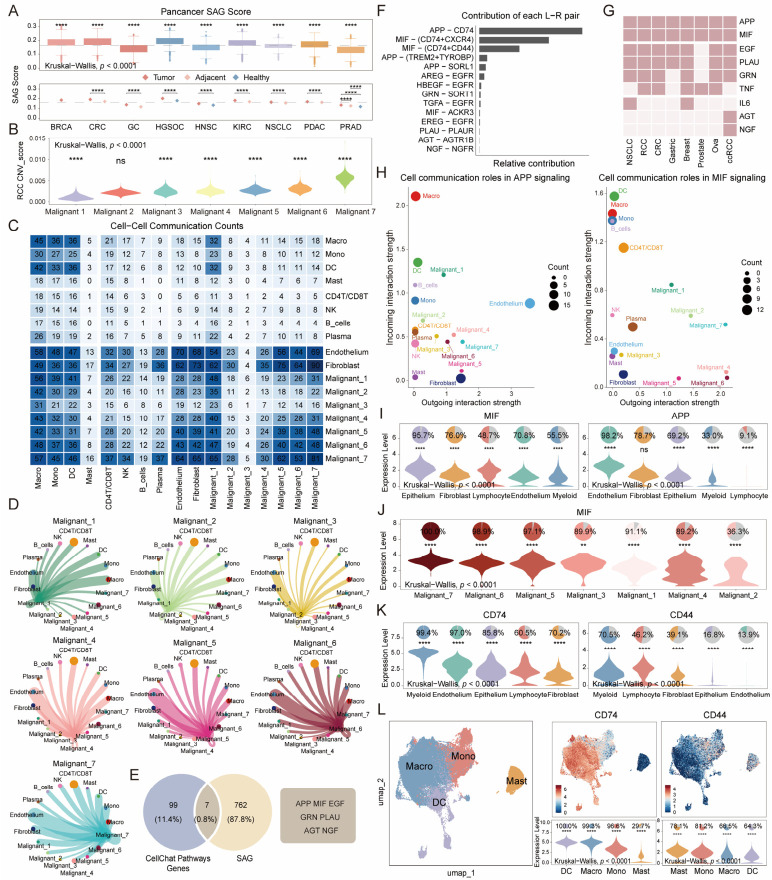
Interaction patterns of senescence-associated cells in the pan-cancer tumor microenvironment: (**A**) SAG scores calculated using the ‘AddModuleScore’ function to systematically assess myeloid cell senescence across cancer types and tissue origins. (**B**) Epithelial cells in RCC were clustered into seven subgroups based on CNV scores and designated Epithelium1–7 according to increasing mean CNV levels. (**C**) Heatmap showing the global matrix of cell–cell communication counts among diverse cell types within the ccRCC tumor microenvironment. (**D**) Circle plots showing intercellular communication networks between seven malignant epithelial cell subpopulations and other cell types, with line thickness proportional to interaction strength. (**E**) Venn diagram showing the intersection between SAGs and key intercellular communication pathway genes identified by CellChat in ccRCC, used to identify senescence-associated pathway genes. (**F**) Signaling pathways involving senescence-related interaction genes in ccRCC. (**G**) Pan-cancer conserved senescence-related interaction genes. (**H**) Intercellular communication roles of different cell types in the *APP* and *MIF* signaling pathways in ccRCC. The *x*-axis denotes outgoing interaction strength, the *y*-axis incoming interaction strength, and circle size represents the number of interactions. (**I**) Expression levels and distribution of *MIF* and *APP* across different cell types in RCC. (**J**) Expression and distribution of *MIF* across malignant epithelial subclusters (Epithelium1–7) in breast cancer. (**K**) Expression and distribution of the senescence-related receptor genes *CD74* and *CD44* across cell types in RCC. (**L**) UMAP of pan-cancer myeloid cells and the expression and distribution of *CD74* and *CD44* at the pan-cancer level. The statistical difference was analyzed by Wilcoxon rank-sum tests with Benjamini–Hochberg adjustment, where **** represents adjusted *p* < 0.0001, ** represents adjusted *p* < 0.01, and ns represents not significant.

We next assessed myeloid cell senescence across healthy, adjacent non-tumor, and tumor tissues at the pan-cancer level. Quantitative analyses showed that, despite inter-cancer heterogeneity, myeloid SAG scores were significantly higher in tumor and adjacent tissues than in healthy counterparts (Figure 2A). This enrichment of senescence-associated features within tumor-associated tissues suggests a potential association between the tumor microenvironment and myeloid cell senescence. Accordingly, these findings raise the possibility that the tumor microenvironment may contribute to the induction of senescence in myeloid cells. To delineate the mechanisms underlying this effect, we first characterized the malignant states of epithelial cells within the TME. Epithelial cells were clustered into seven malignant subgroups (Malignant 1–Malignant 7) based on copy number variations (CNV) scores (Figure 2B and Figure S2A), with the Malignant 7 subgroup exhibiting the highest mean CNV level. We then systematically evaluated intercellular communication patterns across different lineages using CellChat analysis. In clear cell renal cell carcinoma (ccRCC) and multiple other cancer types, the number of ligand-receptor pairs between non-immune cells and myeloid cells was markedly higher than that involving lymphoid cells (Figure 2C and Figure S2B–I). Notably, in gastric cancer (GC), non-small cell lung cancer (NSCLC), ovarian cancer (OV) and ccRCC, higher epithelial malignancy was consistently associated with more intense communication with myeloid cells (Figure 2D and Figure S2B–J).

To identify senescence-related communication events, we intersected key interaction genes identified from CellChat analysis (Supplementary Table S5) with the senescence-associated gene set. In ccRCC, seven senescence-related interaction genes were identified: *APP*, *MIF*, *EGF*, *GRN*, *PLAU*, *AGT*, and *NGF* (Figure 2E). Among these, *APP* was primarily enriched in the *APP*-*CD74* signaling axis, whereas *MIF* was closely associated with both the *MIF*-(*CD74* + *CXCR4*) and *MIF*-(*CD74* + *CD44*) composite signaling pathways (Figure 2F). At the pan-cancer level, *APP* and *MIF* emerged as conserved, high-confidence senescence-associated pathway genes across cancer types (Figure 2G). Importantly, we further delineated the cellular routes of these signals. *APP*-associated senescence signals were predominantly emitted by endothelial cells and targeted macrophages, whereas *MIF*-associated signals originated mainly from malignant epithelial cells and acted primarily on dendritic cells (DCs) and monocytes/macrophages (Mono/Macro) (Figure 2H and Figure S2B–I).

We next systematically examined the expression patterns of senescence-related ligand-receptor genes across cell types at the pan-cancer scale. *APP* expression was largely restricted to endothelial cells, while *MIF* was highly expressed in epithelial cells, with particularly elevated levels in highly malignant epithelial cells (Malignant 7) (Figure 2I,J and Figure S2K). Correspondingly, the receptor genes *CD74* and *CD44* were predominantly enriched in myeloid cells (Figure 2K and Figure S2L). Consistently, spatial transcriptomics confirmed the spatial alignment of MIF expression with epithelial cells and *CD74* with myeloid cells (Figure S2M). These findings indicate pronounced lineage selectivity in senescence signaling, whereby endothelial and epithelial cells preferentially transmit senescence cues to myeloid rather than lymphoid cells.

To further assess myeloid responsiveness to senescence signals, we integrated pan-cancer myeloid single-cell transcriptomic data and visualized the expression of *CD74* and *CD44*. *CD74* showed consistently high expression across DCs, Macro, and Mono, whereas *CD44* displayed more limited expression levels and distribution (Figure 2L). *CD74* therefore represents a principal receptor mediating senescence signal reception in myeloid cells, with Mono, Macro, and DCs constituting the key responsive subpopulations. Collectively, these findings suggest that the myeloid-biased architecture established during physiological aging is preserved in the tumor microenvironment, where senescence-associated signals are preferentially transmitted to myeloid cells through interactions with malignant cells.

### 2.3. Tumor-Associated Myeloid Senescence Core Subpopulation Mac_DAB2 Is Prominently Coupled with Senescence Programs via CD74 Signaling

**Figure 3:**
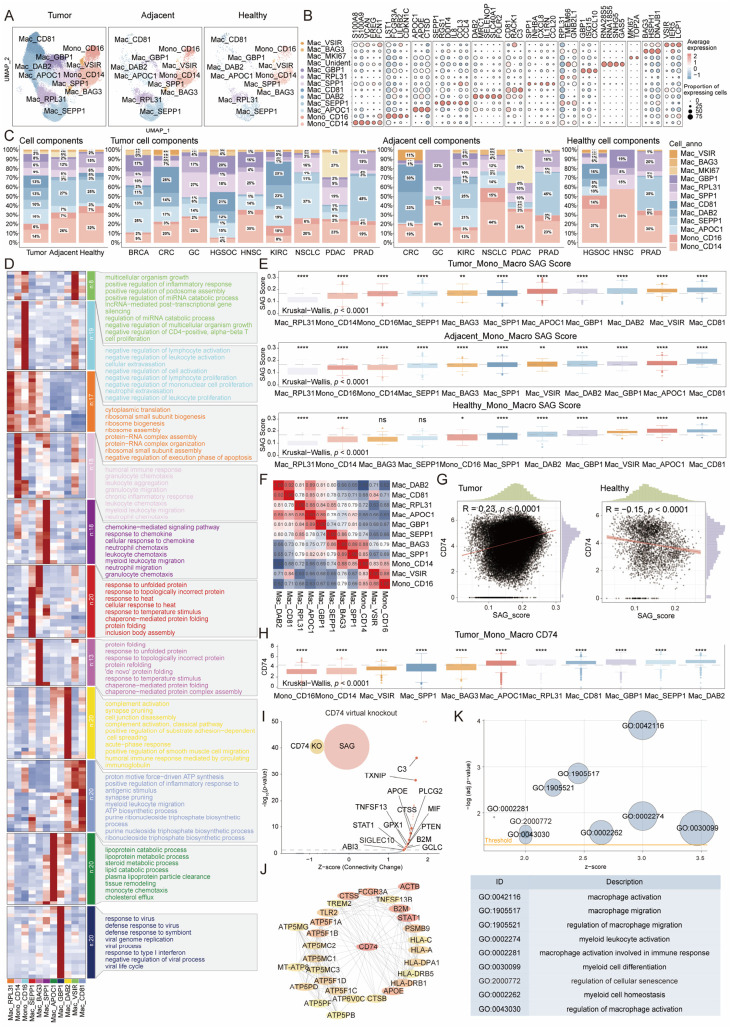
A high-resolution pan-cancer myeloid atlas identifies key senescence-associated subpopulations: (**A**) UMAP of pan-cancer Mono/Macro stratified by tissue origin (Tumor, Adjacent, Healthy). The distribution of 11 core subpopulations is shown; proliferative (*MKI67*^+^) and unclassified (Unident) subsets are excluded from this view. (**B**) Bubble plot showing gene expression patterns across 13 Mono/Macro subpopulations. Dot size indicates the proportion of cells expressing each gene, and color intensity represents relative expression levels. (**C**) Relative abundance of myeloid subpopulations across different tissue microenvironments (All samples, Tumor, Adjacent, Healthy). (**D**) Functional enrichment analysis of 11 myeloid subpopulations in pan-cancer tumor samples. (**E**) Comparison of SAG scores of 11 Mono/Macro subpopulations across Tumor, Adjacent, and Healthy tissues, with subpopulations ordered by increasing mean score. (**F**) Spearman correlation matrix based on transcriptomic profiles of the 11 Mono/Macro subpopulations, with correlation coefficients shown. (**G**) Correlation analysis between cellular SAG scores and *CD74* expression in tumor and healthy tissues. Each dot represents a single cell; red solid lines indicate linear regression fits, and marginal histograms depict data distributions. (**H**) *CD74* expression across myeloid subpopulations in tumor tissues, with subpopulations ranked by ascending mean expression levels. (**I**) Volcano plot showing key affected target genes following *CD74* knockdown in Mac_DAB2 cells. The Venn diagram in the upper left shows the overlap between *CD74* knockdown-affected genes and SAGs, which are highlighted in the volcano plot. (**J**) PPI network of the top 30 *CD74*-regulated downstream genes. Nodes represent individual genes; edges denote interactions. (**K**) Functional enrichment analysis of genes affected by *CD74* knockdown. The statistical difference was analyzed by Wilcoxon rank-sum tests with Benjamini–Hochberg adjustment, where **** represents adjusted *p* < 0.0001, ** represents adjusted *p* < 0.01, * represents adjusted *p* < 0.05, and ns represents not significant.

To identify core myeloid subpopulations that respond to senescence cues and are locked into immune remodeling, we systematically examined changes in myeloid composition within the TME at the pan-cancer level. Among all myeloid lineages, Mono/Macro exhibited the most pronounced abundance reshaping in the TME (Figure S3A,B). High-resolution clustering of the Mono/Macro compartment resolved 11 molecularly distinct subclusters, among which Mac_APOC1, Mac_DAB2, and Mac_CD81 showed consistent and marked enrichment in tumor tissues (Figure 3A–C). Functional enrichment analyses revealed that these three subpopulations undergo a coordinated functional shift from physiological homeostasis toward chronic inflammation and metabolic reprogramming. Specifically, Mac_APOC1 was characterized by lipid metabolic remodeling and tissue remodeling signatures, Mac_DAB2 displayed cascade activation of complement and inflammatory pathways, and Mac_CD81 exhibited a functional phenotype dominated by elevated energy metabolism (Figure 3D and Figure S3C–E). These findings suggest that the expansion of specific myeloid subpopulations within the TME is accompanied by substantial metabolic and inflammatory reprogramming.

To characterize myeloid senescence dynamics at refined subpopulation resolution, we next evaluated SAG scores across Mono/Macro subsets. Mac_CD81 consistently maintained high senescence activity across tissue origins, whereas Mac_DAB2 displayed a stepwise increase in senescence from healthy through adjacent to tumor tissues (Figure 3E). Transcriptomic similarity analysis further revealed a convergence of lineage features between Mac_DAB2 and the highly senescent Mac_CD81 subpopulation (Figure 3F), a pattern not observed in DC subsets (Figure S3F–J).

At the molecular level, the previously identified senescence signal receptor *CD74* exhibited marked microenvironmental dependence. *CD74* expression was negatively correlated with senescence scores under physiological conditions but shifted to a significant positive correlation in tumor tissues (Figure 3G), suggesting reactivation of *CD74*-mediated senescence signaling in pathological contexts. Cross-lineage comparisons further highlighted a selective up-regulation of *CD74* in the Mac_DAB2 subpopulation within the TME, with its expression levels progressively intensifying along the microenvironmental transition (Figure 3H and Figure S3K). *CD74* expression was extensively and positively correlated with canonical senescence markers (*CDKN1A*, *CDKN2A*, *GLB1*, and *TP53*) as well as a broad spectrum of SASP-related genes (Figure S3L). Given the prominent expression of *CD74* and the progressive accumulation of senescence features in Mac_DAB2, we next explored the potential association between *CD74* signaling and senescence-related transcriptional programs in this subpopulation. To this end, we performed in silico knockdown of *CD74* specifically within the Mac_DAB2 cluster under TME conditions. We observed a significant enrichment of senescence-associated genes among the top-ranked perturbed candidates (Figure 3I). Notably, *CD74* deficiency markedly disrupted myeloid immune checkpoint genes, particularly *SIGLEC10*. Correlation analysis further revealed that *CD74* expression and senescence program activation were tightly coupled with the coordinated upregulation of canonical immune checkpoints, including *SIRPA*, *LILRB1*, and *SIGLEC10*, exhibiting a stable co-linearity specifically within tumor tissues (Figure S3M). Furthermore, protein–protein interaction (PPI) analysis of the top 30 affected genes revealed that these senescence-related downstream targets occupied central hub positions within the regulatory network (Figure 3J). Subsequent functional enrichment analysis showed that genes affected by *CD74* perturbation were significantly enriched in senescence-related regulatory pathways in Mac_DAB2 cells (Figure 3K). Spatial deconvolution analysis suggested potential spatial co-localization among *MIF*-expressing epithelial cells, *CD74* expression, and the Mac_DAB2 subpopulation (Figure S3N). Collectively, these findings identify Mac_DAB2 as a core tumor-associated senescent myeloid subpopulation and implicate *CD74* as a potential receptor involved in senescence-associated signaling.

### 2.4. The Tumor Microenvironment Hijacks a Conserved Myeloid Senescence-Associated Gene Signature (MSAG) Originating from Physiological Differentiation

**Figure 4:**
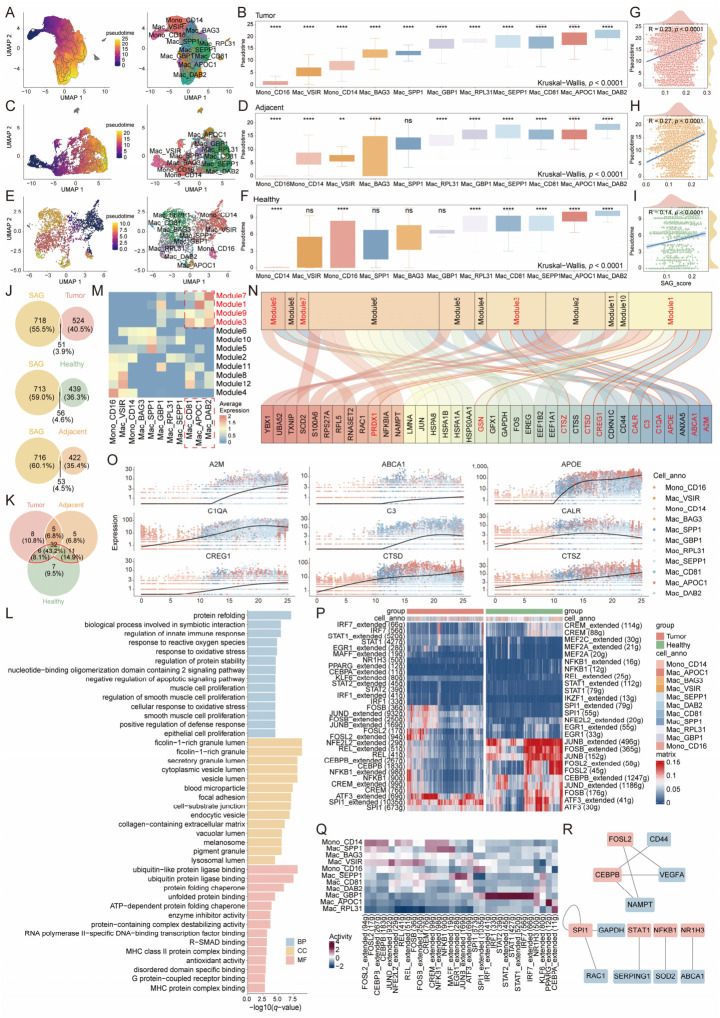
Dynamic accumulation of Mono/Macro senescence programs along differentiation trajectories and their transcriptional regulatory basis: (**A**) Inferred differentiation trajectories of Mono/Macro subpopulations in tumor tissues. In the UMAP, cells are colored by pseudotime (left) or by cell type (right), with the 11 Mono/Macro subpopulations annotated. (**B**) Mean pseudotime values of Mono/Macro subpopulations in tumor tissues, ordered from low to high. (**C**,**D**) UMAP of Mono/Macro differentiation trajectories in adjacent tissues and ranking of subpopulations by mean pseudotime. (**E**,**F**) UMAP of Mono/Macro differentiation trajectories in healthy tissues and ranking of subpopulations by mean pseudotime. In panels (**A**,**C**,**E**), the numbers indicate the root states used to initiate pseudotime ordering. (**G**–**I**) Spearman correlation analyses between senescence scores and pseudotime values of Mono/Macro cells in tumor, adjacent, and healthy tissues. (**J**) Intersection analysis between SAGs and trajectory-associated genes of Mono/Macro cells in tumor, adjacent, and healthy tissues; the number of overlapping genes in each group is indicated. (**K**) Intersection analysis of trajectory-related senescence driver genes across tissue origins, highlighting 49 core genes shared between healthy tissues and tumor/adjacent tissues. (**L**) Functional enrichment analysis of the 49 intersecting genes identified in healthy tissues. (**M**) Heatmap of gene co-expression modules in Mono/Macro subpopulations from tumor tissues, with colors indicating average module expression across subpopulations. (**N**) Sankey diagram showing the distribution of senescence key genes across co-expression modules in tumor tissues. (**O**) Dynamic expression patterns of senescence key genes along pseudotime across Mono/Macro subpopulations. (**P**) Heatmap of inferred regulon activities in Mono/Macro subpopulations from tumor and healthy tissues. Columns are annotated by tissue origin and cell subpopulation; rows represent differentially active regulons, and colors indicate normalized regulon activity levels. (**Q**) Regulon activity levels across Mono/Macro subpopulations in tumor tissues. (**R**) Transcriptional regulatory network linking core transcription factors and senescence key genes in tumor tissues. Red nodes denote core transcription factors, and blue nodes represent their downstream senescence-associated targets. The statistical difference was analyzed by Wilcoxon rank-sum tests with Benjamini–Hochberg adjustment, where **** represents adjusted *p* < 0.0001, ** represents adjusted *p* < 0.01, and ns represents not significant.

Trajectory inference analyses were performed on Mono/Macro cells from tumor, adjacent, and healthy tissue microenvironments to characterize the evolution of senescence states along cellular differentiation. In all tissue contexts, Mac_APOC1, Mac_CD81, and Mac_DAB2 localized to the terminal ends of pseudotime trajectories, with Mac_DAB2 exhibiting a consistent terminal distribution across tissues (Figure 4A–F). Further analyses revealed a significant positive correlation between senescence scores and pseudotime progression, with steeper increases in senescence scores per unit pseudotime observed in tumor and adjacent tissues compared with healthy tissues (Figure 4G–I).

Trajectory-associated genes of Mono/Macro differentiation were identified and intersected with the senescence-associated gene set. This analysis yielded 51, 56, and 53 trajectory-related senescence-associated genes in tumor, adjacent, and healthy tissues, respectively (Figure 4J). To define the core molecular components of myeloid senescence, we intersected trajectory-related senescence key features identified in healthy tissues with those from tumor and adjacent tissues, resulting in a set of 49 senescence key genes (Figure 4K). Functional enrichment analysis of these genes indicated predominant involvement in proteostasis and protein folding, oxidative stress responses, immune regulation and antigen presentation, as well as secretory/lysosomal pathways and cell adhesion processes (Figure 4L). Using trajectory-associated genes, we constructed gene co-expression modules and examined their expression patterns across Mono/Macro subpopulations. In tumor tissues, Mac_APOC1, Mac_CD81, and Mac_DAB2 showed marked upregulation of Module7, Module1, Module9, and Module3 (Figure 4M). We further assessed the distribution and expression of senescence key genes within modules in tumor (38 genes), adjacent (43 genes), and healthy (49 genes) tissues. Across all tissue contexts, senescence key genes within modules upregulated in Mac_APOC1, Mac_CD81, and Mac_DAB2 exhibited progressively increased expression along pseudotime (Figure 4N,O and Figure S4A–D). These findings indicate that senescence programs progressively accumulate along myeloid differentiation trajectories, peaking in terminal subpopulations.

To explore potential upstream regulatory programs associated with myeloid senescence, we inferred transcription factor regulons in tumor, adjacent, and healthy tissues. Tumor and adjacent tissues exhibited a greater number of highly active regulons than healthy tissues, whereas the average activity of dominant regulons was relatively higher in healthy tissues (Figure 4P and Figure S4E). Subpopulation-specific regulon activity patterns suggested preferential associations of *NR1H3* with Mac_APOC1, whereas *ATF3* emerged as a prominent candidate regulator shared by Mac_CD81 and Mac_DAB2 within the TME. Notably, candidate transcription factors of macrophage subpopulations differed substantially across tissue microenvironments, indicating pronounced context-dependent regulatory heterogeneity (Figure 4Q and Figure S4F–J). Finally, we constructed transcriptional regulatory networks linking candidate transcription factors to senescence key genes across tissue contexts, highlighting potential regulatory programs associated with myeloid senescence (Figure 4R and Figure S4K,L).

### 2.5. MSAG Defines an Immunosuppressive, High-Risk Senescent Subtype

**Figure 5:**
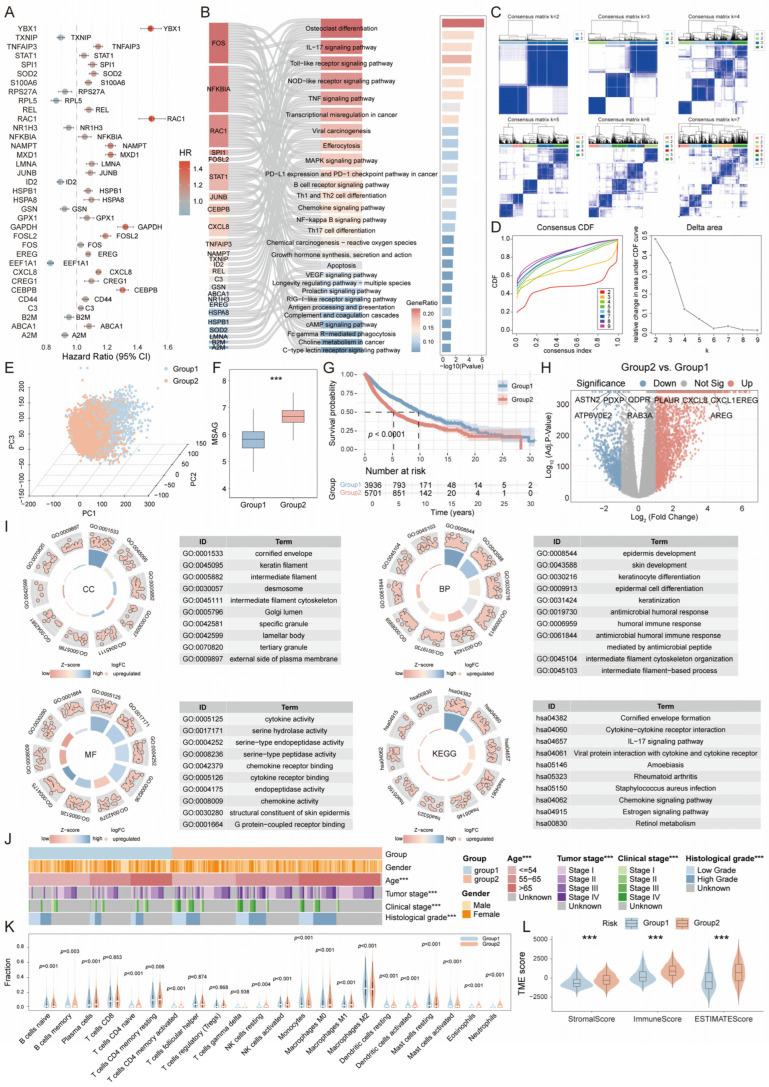
MSAG-defined myeloid senescence molecular subtypes and their clinical and immunological features: (**A**) Forest plot showing univariate Cox regression results for 35 constituent genes of MSAG.SIG, with dots indicating HRs. (**B**) Sankey-bar plot from KEGG enrichment analysis showing the associations between MSAG.SIG and significantly enriched pathways. The bar chart on the right indicates pathway enrichment significance, with colors corresponding to the GeneRatio. (**C**) Consensus matrix derived from consensus clustering based on the MSAG.SIG expression profiles. (**D**) CDF curves and corresponding delta area plots for consensus clustering. (**E**) Three-dimensional PCA scatter plot showing the distribution of two myeloid senescence subtypes along the first three principal components (PC1, PC2, PC3). (**F**) Box plots comparing the overall MSAG.SIG expression levels between the two subtypes; statistical significance was assessed using the Wilcoxon rank-sum test. (**G**) Kaplan–Meier survival curves showing significant stratification of overall survival between the two molecular subtypes; shaded areas denote 95% confidence intervals, and significance was determined by the log-rank test. (**H**) Volcano plot depicting differentially expressed genes between group 2 and group 1; red and blue dots represent significantly upregulated and downregulated genes, respectively, with the top five most significant genes labeled on each side. (**I**) Donut chart showing the top 10 significantly enriched biological functions and pathways among the differentially expressed genes. (**J**) Clinical feature heatmap illustrating differences between the two subtypes in key pathological parameters, including gender, age, tumor stage, clinical stage, and histological grade; significance was assessed using the chi-square test. (**K**) Violin plots showing the estimated infiltration abundance of 22 immune cell types within each group based on the ‘CIBERSORT’ algorithm. (**L**) Comparison of TME scores between the two subtypes, including stromal score, immune score, and ESTIMATE score. The statistical differences were analyzed by Wilcoxon rank-sum tests (*** represents adjusted *p* < 0.001), whereas differences shown in (**J**) were evaluated using Pearson’s chi-squared tests (*** represents *p* < 0.001).

To evaluate the robustness of the 49 senescence-associated genes and their 10 core transcription factors identified at the single-cell level in bulk tissues, we mapped these 59 candidates to the TCGA pan-cancer cohort and assessed their prognostic relevance. Univariate Cox regression identified 35 genes significantly associated with overall survival across cancers, which were defined as the myeloid senescence-associated prognostic signature (MSAG.SIG) (Figure 5A). Functional enrichment analysis revealed that these genes were primarily involved in myeloid cell differentiation and responses to reactive oxygen species and hydrogen peroxide, canonical processes linked to senescence induction. KEGG pathway analysis further showed significant enrichment not only in pro-inflammatory signaling pathways such as *NF-κB*, *TNF*, and *IL-17*, but also in the *PD-L1* expression and *PD-1* checkpoint pathway and longevity-regulating pathways (Figure 5B and Figure S5A). These results suggest that MSAG.SIG is associated with transcriptional programs linked to inflammation and immunosuppression.

The molecular subtyping potential of MSAG.SIG at the pan-cancer level was assessed by performing unsupervised consensus clustering on the TCGA cohort using the expression profiles of its 35 constituent genes. Integrated evaluation of the cumulative distribution function (CDF) curves, delta area changes, and consensus matrix stability identified k = 2 as the optimal solution, yielding two molecular subtypes designated group 1 and group 2 (Figure 5C,D). Principal component analysis demonstrated clear transcriptomic separation between the two subtypes, supporting the robustness of this classification (Figure 5E). Comparative analysis showed that group 2 exhibited significantly higher overall MSAG.SIG scores (Figure 5F). Survival analysis further revealed poorer overall survival in group 2 patients (Figure 5G), indicating a close association between activation of the myeloid senescence program and adverse clinical outcomes. To explore the mechanisms underlying the prognostic divergence, we analyzed differentially expressed genes between the two groups. Genes upregulated in group 2 were significantly enriched in differentiation- and inflammation-related pathways, which may constitute the biological basis driving tumor progression and unfavorable prognosis in this subtype (Figure 5H,I).

Clinical characterization further highlighted the distinct aging and malignant features of group 2. This subtype was enriched for patients older than 65 years and showed a higher prevalence of advanced tumor stage and high histological grade, confirming its strong association with adverse clinicopathological features (Figure 5J). In terms of TME composition, group 2 displayed a myeloid-dominant infiltration pattern accompanied by significantly elevated stromal and immune scores (Figure 5K,L). This subtype was characterized by concurrent stromal enrichment and myeloid infiltration, features commonly associated with an immunosuppressive tumor microenvironment. Consistently, group 2 exhibited marked upregulation of immune checkpoint genes, suggesting a more immunosuppressive microenvironment (Figure S5B). Collectively, MSAG.SIG effectively identifies a high-risk patient subset at the pan-cancer scale. This group exhibits distinct hallmarks, including senescence-associated myeloid remodeling, aggressive clinicopathological features, and potential resistance to immunotherapy. Such robust correlation underscores its value for precise risk assessment.

### 2.6. An MSAG-Based Scoring Model Enables Precise Pan-Cancer Risk Stratification and Survival Prediction

**Figure 6:**
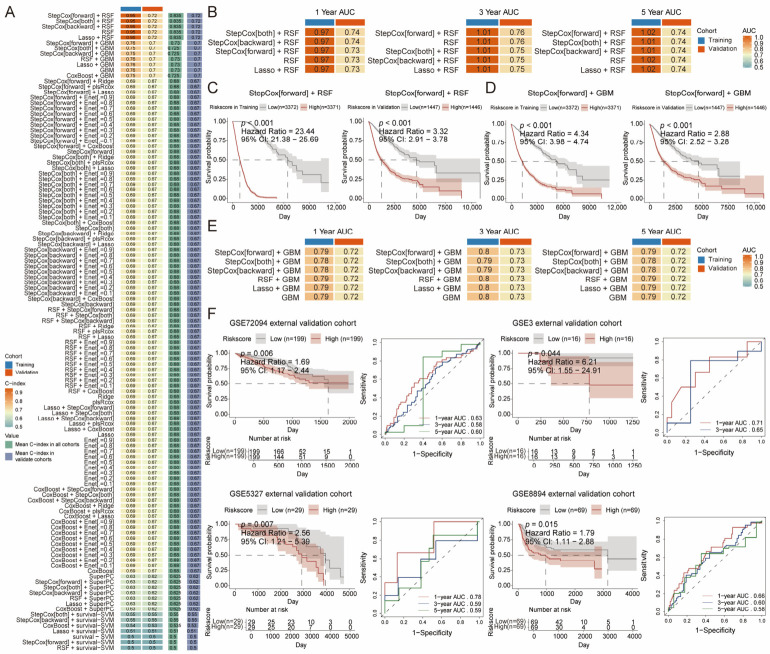
Construction and cross-cohort validation of the MSAG.SIG-based prognostic model: (**A**) Integrated C-index performance of 117 machine-learning combination models in the TCGA training cohort (*n* = 6743) and internal validation cohort (*n* = 2893). (**B**) AUC values at 1, 3, and 5 years for the StepCox[forward] + RSF model in the training and internal validation cohorts. (**C**) Kaplan–Meier survival analysis of the StepCox[forward] + RSF model in the training and internal validation cohorts. (**D**) Kaplan–Meier survival analysis of the StepCox[forward] + GBM model in the training and internal validation cohorts. (**E**) AUC values at 1-, 3-, and 5-year for the StepCox[forward] + GBM model in the training and internal validation cohorts. (**F**) Kaplan–Meier survival analyses and corresponding time-dependent ROC curves for the StepCox[forward] + GBM model in multiple independent external validation cohorts.

The translational potential of MSAG.SIG for prognostic prediction was systematically evaluated by randomly splitting the TCGA pan-cancer transcriptomic cohort into a training set and an internal validation set at a 7:3 ratio. The 35 constituent genes were used as input features to construct 117 prognostic models by integrating 10 machine-learning algorithms (Figure 6A). Initial C-index assessment revealed that five random survival forest (RSF)-based combinations exhibited abnormally high fitting performance in the training set, with 1-, 3-, and 5-year area under the curve (AUC) values and hazard ratios suggesting a high risk of overfitting (Figure 6B,C and Figure S6A). We therefore focused on four alternative combinations, including StepCox[forward] + GBM, which maintained high predictive accuracy while showing consistent AUC performance between training and validation cohorts. StepCox[forward] + GBM was ultimately selected as the optimal MSAG.SIG prognostic model (Figure 6D,E and Figure S6B). The clinical utility of the final model was further validated in independent external gene expression omnibus (GEO) cohorts. Risk scores derived from this model effectively stratified patients into high- and low-risk groups across multiple cancer types and retained stable AUC values for 1-, 3-, and 5-year survival prediction (Figure 6F). Together, these results demonstrate that the MSAG.SIG-based prognostic score not only performs robustly in the training cohort but also generalizes well to independent external datasets, supporting myeloid senescence features as pan-cancer prognostic biomarkers with translational relevance.

## 3. Discussion

Physiological aging is associated with a myeloid-dominated immune infiltration pattern in normal tissues, reflecting disrupted immune homeostasis under chronic inflammation rather than enhanced immunity [25]. Our analyses suggest that the myeloid-biased immune state is established during physiological aging and is also observed in tumor tissues. Tumors may therefore retain and further accentuate pre-existing senescence-associated immune features of the host, with myeloid cells acting as a central bridge between aging and the tumor microenvironment. Understanding this process may help explain the association between aging and tumor progression and provide a framework for prognostic evaluation and therapeutic exploration in cancer.

Although myeloid cells are the main carriers of immune aging in both physiological and pathological conditions, myeloid senescence scores are significantly higher in tumors than in healthy tissues. Since malignant components distinguish tumors from normal tissues, we hypothesize that aberrant microenvironmental signaling underlies this effect. Intercellular communication analyses supported this possibility, revealing a consistent, lineage-specific pattern of senescence signaling that preferentially targets myeloid cells. Malignant and endothelial cells were predicted to preferentially communicate with myeloid cells through senescence-associated signaling pathways, a pattern that may contribute to the elevated senescence features observed in these populations. Further analyses suggested that interactions between tumor cells and myeloid populations are accompanied by progressive changes in senescence-associated transcriptional programs. This qualitative reprogramming is exemplified by our in-depth analysis of *CD74*. Identified through ligand–receptor interaction analyses as a candidate receptor associated with senescence-related signaling, *CD74* exhibits a striking environment-dependent functional inversion. Under healthy physiological conditions, *CD74* expression is negatively correlated with senescence scores, which is consistent with a homeostatic role in restraining excessive senescence. In tumor tissues, however, a significant positive correlation is observed. Although these observations do not establish a causal role for *CD74*, they suggest a potential association between *CD74* signaling and senescence-associated, inflammatory, and immunoregulatory programs within the tumor microenvironment.

We next focused on the myeloid lineage to identify myeloid subpopulations most strongly associated with senescence-related features. Through refined subclustering and functional annotation, we identified a characteristic Mac_DAB2 population. Functional enrichment analyses suggest that this subset is characterized by distinct metabolic features. Specifically, it is enriched in pathways related to glycosaminoglycan (GAG) degradation and lipid metabolism, including lipoprotein clearance and arachidonic acid metabolism, accompanied by coordinated upregulation of inflammatory modules such as complement activation. Previous studies have shown that active arachidonic acid and sphingolipid metabolism is not merely a source of energy, but a prerequisite for the synthesis of lipid-based SASP factors, such as prostaglandins, that directly drive chronic inflammation [26,27,28]. In parallel, enhanced GAG metabolism may be indicative of extracellular matrix remodeling, the degradation products of which often act as DAMPs that further activate immune receptors [29,30].

In addition, senescence scores show a strong positive correlation with the expression of the inhibitory receptor *SIGLEC10* on macrophages. Given that *SIGLEC10* functions as a receptor for the *CD24*-mediated ‘don’t eat me’ signal [31], the positive association between senescence scores and *SIGLEC10* expression raises the possibility that senescent macrophages may be linked to altered phagocytic regulatory programs. Together, these findings suggest that senescent myeloid cells are not simply functionally exhausted, but are reprogrammed through coupled metabolic and immune mechanisms to construct an immune-evasive niche that protects tumors.

Whereas the above findings characterize the cellular and microenvironmental features associated with immune aging, we next explored the transcriptional programs potentially linked to its maintenance. Pseudotime analyses revealed a tight coupling between cellular senescence and monocyte-to-macrophage differentiation. Across healthy, adjacent, and tumor tissues, senescence scores progressively accumulate along the differentiation trajectory and peak within the terminal Mac_DAB2 population. Compared with the gradual, homeostatic progression observed in healthy tissues, that in tumor and adjacent tissues exhibits a much steeper slope of senescence accumulation. Together, these observations suggest that myeloid differentiation remains largely conserved across tissue contexts, while senescence-associated programs are enhanced in tumor-associated environments.

Based on this ‘physiology as foundation, pathology as outcome’ framework, we adopted a distinctive physiologically anchored strategy to define a core senescence gene signature. We first identified baseline gene sets involved in normal aging-differentiation processes in healthy tissues, and then intersected these with key genes from tumor and adjacent tissues. Notably, the overlapping gene set accounted for the vast majority of senescence-associated genes, whereas tissue-specific genes unique to the tumor microenvironment constituted only a minor fraction, as illustrated by the Venn diagram. This substantial overlap suggests that many senescence-associated features observed in tumor tissues are shared with physiological aging programs, whereas a smaller subset appears to be tissue-context specific. Accordingly, the intersecting gene set defined as MSAG may represent a conserved transcriptional signature shared between physiological and pathological senescence contexts. These genes are shared between physiological aging and tumor-associated senescence, and exhibit increased expression within tumor tissues. Importantly, inferred regulatory network analyses suggested coordinated activation of MSAG-associated genes toward the terminal stages of the pseudotime trajectory.

Finally, we extended our perspective from single-cell-resolved mechanisms to the population scale of pan-cancer cohorts to evaluate the translational potential and prognostic robustness of MSAG. Consensus clustering based on MSAG.SIG identified a high-risk subtype characterized by aging-associated and highly malignant features. This subtype is enriched for older patients at the demographic level and exhibits advanced tumor stage and grade at the pathological level. The association between elevated MSAG.SIG activity and poor clinical outcomes suggests that myeloid senescence-related programs may be linked to adverse prognostic features. The ‘high-stroma-high-myeloid’ infiltration pattern observed in group 2 reveals the microenvironmental basis of its adverse outcome. Dense stromal deposition together with increased myeloid infiltration may contribute to an immune-restrictive microenvironment. Collectively, MSAG.SIG may serve as a prognostic indicator and provide a framework for exploring senescence-associated therapeutic strategies. For patients who derive limited benefit from immune checkpoint blockade alone, combinatorial strategies targeting myeloid senescence features—such as disrupting metabolic reprogramming or blocking senescence signal transmission—may represent a key avenue to improve clinical outcomes. The prognostic model established within a rigorous machine-learning framework and validated across independent cohorts not only confirms the robustness and generalizability of MSAG as a pan-cancer biomarker, but also provides a solid translational foundation for converting the complex biology of myeloid immune senescence into a quantitative stratification tool for precision oncology.

We acknowledge several limitations of this study. First, although the integration of single-cell transcriptomics, spatial co-localization, and in silico knockouts suggests a potential pathway where malignant epithelial cells transmit senescence signals to the Mac_DAB2 myeloid subpopulation via the *MIF*-*CD74* axis, the exact causal relationship between myeloid senescence and tumor progression remains to be conclusively established. Additionally, the precise co-localization patterns of these cells under real biological conditions warrant further validation through in vitro and in vivo functional experiments. Second, the inferred upstream transcriptional regulatory networks rely primarily on probabilistic predictions derived from static co-expression matrices. Such static models cannot accurately capture dynamic biochemical processes, such as the functional nuclear localization or phosphorylation-induced activation of transcription factors. Therefore, these regulatory mechanisms should be cautiously interpreted as predicted programs, and their precise biochemical wiring remains to be elucidated by direct biological evidence in future studies.

## 4. Materials and Methods

### 4.1. GTEx Normal Tissue Data

Normal tissue transcriptomic data and corresponding age information were obtained from GTEx (https://gtexportal.org/home/, accessed on 17 September 2025), covering organs corresponding to 17 common cancer types: breast, prostate, lung, liver, colon, stomach, esophagus, pancreas, cervix uteri, uterus, ovary, skin, thyroid, bladder, kidney, brain, and small intestine. Samples covered ages 20–79 years and were grouped into young (20–39 years) and aged (40–79 years) cohorts.

Enrichment of cell type-specific gene signatures was quantified using single-sample gene set enrichment analysis (ssGSEA). To account for scale differences across cell types, enrichment scores were normalized within each cell category. The analyzed cell types and their corresponding marker gene sets are provided in Supplementary Table S1.

### 4.2. Senescence-Associated Gene Identification

SAGs were curated from multiple publicly available databases and literature sources, including Aging Map, MSigDB (search terms: ‘senescence’ and ‘aging’), CellAge (filtered for genes annotated as having a ‘Senescence Effect’ of ‘induces’), GenAge, CSGene, as well as several published studies (Supplementary Table S2) [32,33,34,35,36]. After removal of redundant entries, the resulting non-overlapping gene set was used for downstream senescence scoring in myeloid and lymphoid immune cells. Based on the normalized single-cell gene expression matrix, senescence scores were calculated at the single-cell level using the ‘AddModuleScore’ function implemented in the R ‘Seurat’ package.

### 4.3. Single-Cell Dataset Acquisition

To investigate cellular senescence at single-cell resolution, we collected ten scRNA-seq datasets from the GEO (https://www.ncbi.nlm.nih.gov/geo/, accessed on 19 April 2024). These datasets encompass nine common solid tumor types: BRCA (Breast carcinoma, GSE176078), RCC (Renal cell carcinoma, GSE159115), ccRCC (Clear Cell Renal Cell Carcinoma, GSE207493), CRC (Colorectal cancer, GSE166555), GC (Gastric carcinoma, GSE167297), HNSCC (Head and neck squamous cell carcinoma, GSE139324), PDAC (Pancreatic ductal adenocarcinoma, GSE205049), NSCLC (Non-small-cell lung cancer, GSE117570), OV (Ovarian cancer, GSE184880), and PRAD (Prostate adenocarcinoma, GSE181294). In total, these datasets comprised 206 samples, including tumor tissues, adjacent non-tumor tissues, and healthy controls. To reduce batch effects from different sequencing platforms, only datasets generated using the 10× Genomics Chromium platform were included.

### 4.4. ScRNA-Seq Preprocessing and Cell Type Annotation

All single-cell datasets were processed using the ‘Seurat’ package (version 4.4.0) for quality control and normalization. Low-quality cells, defined as nFeature_RNA < 200 or percent.mt > 10%, were excluded. Remaining cells were normalized, centered, and scaled. Subsequently, principal component analysis (PCA) was performed on highly variable genes to extract primary low-dimensional features, which were then used for dimensionality reduction and clustering analysis. Batch effects across samples were minimized using the ‘Harmony’ algorithm. The resulting batch-corrected embedding was used for downstream analyses, including cell clustering, visualization using Uniform Manifold Approximation and Projection (UMAP), and cell type annotation.

Cell annotation employed a three-round hierarchical annotation strategy to enhance precision and ensure consistency across samples and cancer types. In the first round, clustering identified five major cell types: Myeloid, Lymphocyte, Endothelium, Fibroblast, and Epithelium. In the second round, Myeloid and Lymphoid cells were subsetted for further subtype classification using established marker genes: Monocytes (Mono), Macrophages (Macro), DCs, Mast cells, CD4/CD8 T cells, NK cells, B cells, and Plasma cells. In the third round, dimensionality reduction, clustering, and marker gene validation were repeated on the subsetted populations. Cells that did not match subgroup characteristics were removed. This iterative refinement yielded high-confidence cell annotations. After quality control and annotation, a total of 685,541 high-quality cells were retained for downstream analyses of immune senescence. Single-cell metabolic activity was subsequently evaluated using the ‘scMetabolism’ package (version 0.2.1).

### 4.5. Malignant Cell Identification

Malignant cells within epithelial populations were identified using the ‘InferCNV’ R package (version 1.22.0) to infer chromosomal CNV from single-cell transcriptomes. Expression matrices from tumor samples across multiple datasets (GSE176078, GSE207493, GSE166555, GSE167297, GSE117570, GSE184880, GSE181294, GSE159115) were used. Lymphocytes and myeloid cells were used as the reference sets, while epithelial cells were treated as the observation set to generate a CNV expression matrix. K-means clustering (k = 7) was subsequently applied to this matrix, and cells were stratified based on inferred CNV scores to distinguish malignant from non-malignant epithelial states.

### 4.6. Cell–Cell Communication Network Analysis

Cell–cell communication within the TME was inferred from Seurat-processed and normalized single-cell gene expression matrices. Intercellular communication analysis was performed using the ‘CellChat’ package (version 2.1.2), with CellChatDB.human as the reference ligand-receptor interaction database. Three signaling categories were included: secreted signaling, extracellular matrix-receptor interactions, and direct cell–cell contact. The computation of cell communication probabilities was performed using the ‘ComputeCommunProb’ function, employing the ‘TriMean’ method for robust estimation of ligand-receptor expression levels. Cell groups containing fewer than ten cells were excluded to reduce noise from low-abundance populations. Pathway-level communication was inferred using the ‘ComputeCommunProbPathway’ function by integrating ligand-receptor pairs within the same signaling pathway. Global intercellular communication networks were constructed using the ‘AggregateNet’ function.

### 4.7. Spatial Transcriptomics Data Analysis and Deconvolution

To evaluate spatial cell-type compositions and co-localization, we collected four spatial transcriptomics datasets from the European Bioinformatics Institute (EBI, https://www.ebi.ac.uk/, accessed on 11 June 2026) and GEO. These datasets include E-MTAB-12767, GSE251950, GSE175540, and GSE211956. All datasets were analyzed using the ‘Seurat’ package (version 4.4.0). To determine the cell-type composition of each cluster, reference-based deconvolution was performed via the ‘CARD’ package (version 1.1), leveraging scRNA-seq reference data of key cancer subtypes (Epithelium, Macro, MIF_Epi, and DAB2_Mac). Finally, hallmark genes were overlaid via the ‘SpatialFeaturePlot’ function, and spatial proportions of target cell populations were mapped using the ‘CARD’ package to evaluate their spatial co-localization.

### 4.8. In Silico Knockout Analysis

To evaluate the role of *CD74* in driving the senescence phenotype of the Mac_DAB2 subset within the tumor microenvironment, we performed an in silico knockdown of *CD74* in DAB2_Mac tumor cells using the R package ‘scTenifoldKnk’ (v1.0.3), with a q-value cutoff of 0.05 to identify downstream genes significantly affected by the knockdown.

### 4.9. Protein–Protein Interaction Network Analysis

Downstream genes significantly affected by the *CD74* in silico knockdown (*p* < 0.05) were subjected to PPI analysis using the STRING database. The resulting PPI network was visualized with Cytoscape (version 3.7.2), and the functional roles and centrality of individual genes within the transcriptional regulatory network were further assessed using the cytoHubba plugin with the maximum neighborhood component (MNC) algorithm. The top 30 nodes ranked by MNC were designated as core hub genes to elucidate downstream regulatory relationships mediated by *CD74*.

### 4.10. Monocyte-Macrophage Trajectory Analysis

Mono/Macro lineage trajectories were reconstructed using the R package ‘Monocle3’ (version 1.3.7). Proliferative Mac_MKI67 cells and unannotated Mac_Unident cells were excluded prior to analysis, and the remaining 11 Mono/Macro subclusters were retained. A cell data set (CDS) object was constructed from normalized gene expression matrices together with corresponding cell and gene annotations. Dimensionality reduction and clustering were performed using the top 30 principal components. Samples were aligned in low-dimensional space using the ‘align_cds’ function, followed by nonlinear dimensionality reduction and visualization using UMAP. Trajectory topology was inferred using the graph-learning algorithm implemented in ‘learn_graph’, with parameters set to minimal_branch_len = 20 and euclidean_distance_ratio = 10. The Mono_CD14 subcluster was specified as the root state, and pseudotime-ordering was performed using the ‘order_cells’ function. Genes showing dynamic expression changes along pseudotime were identified using Moran’s I spatial autocorrelation statistic, with a significance threshold of q < 0.05.

### 4.11. Transcriptional Regulatory Network Inference

Single-cell transcriptional regulatory networks were inferred using the R package SCENIC (v1.3.1). Transcription factor (TF)-target co-expression networks were inferred using the ‘GENIE3’ algorithm. These networks were subsequently pruned using ‘RcisTarget’ together with the hg19 cis-regulatory motif database (covering TSS ±5 kb and 500 bp upstream regions), retaining the top 5% of target genes per TF to define high-confidence regulons. Regulon activity at the single-cell level was quantified using AUCell. Regulon specificity scores (RSS) were then applied to identify and rank key driver TFs specific to individual Mono/Macro subclusters. TF-target regulatory pairs identified by SCENIC were extracted, and only high-confidence interactions supported by motif annotation and with a Spearman correlation coefficient > 0.2 were retained.

### 4.12. Bulk RNA-Seq Data Collection

Pan-cancer transcriptomic data and corresponding clinical information were obtained from the UCSC Xena website (https://xenabrowser.net/datapages/, accessed on 5 July 2025), including TCGA Pan-Cancer RNA-seq data (Pan-cancer RSEM-FPKM) covering 33 cancer types. Ensembl gene identifiers were converted to gene symbols using the official annotation file. For genes with multiple transcripts, expression values were summarized at the gene level by averaging transcript-level expression. The final dataset contained expression profiles of 18,338 protein-coding genes across 10,535 samples and was used for subsequent pan-cancer analyses. In addition, publicly available bulk RNA-seq datasets from the GEO, including GSE72094, GSE5327, GSE3, and GSE8894, were collected as external validation cohorts.

### 4.13. Consensus Clustering of Myeloid Senescence Genes

Unsupervised clustering of prognostically relevant myeloid senescence gene expression profiles in the TCGA cohort was performed using the ‘ConsensusClusterPlus’ package (version 1.70.0). Clustering was carried out using the partitioning around medoids (PAM) algorithm with Euclidean distance. To evaluate clustering stability across different numbers of clusters (K), resampling was conducted over 100 iterations, with 80% of samples and 100% of genes randomly selected in each iteration.

### 4.14. Immune Infiltration and TME Analysis

Tumor immune cell infiltration was estimated from TCGA Pan-Cancer RNA-seq data using the ‘CIBERSORT’ algorithm. Global TME characteristics were further quantified using the ‘ESTIMATE’ algorithm to calculate StromalScore, ImmuneScore, and ESTIMATEScore, which were used to compare differences in microenvironmental composition between groups.

### 4.15. Pan-Cancer Prognostic Model Construction

TCGA Pan-Cancer transcriptomic and corresponding clinical data were integrated to construct a comprehensive dataset comprising 9636 samples. The dataset was randomly split into training and validation sets in a 7:3 ratio, with a fixed random seed to ensure reproducibility. Candidate prognostic genes were screened by univariate Cox regression analysis (*p* < 0.05) to generate a gene list for subsequent machine learning-based model construction (Supplementary Table S3). A multi-algorithm ensemble strategy was applied to the training and validation sets to build pan-cancer prognostic models, with cross-validation used to select the optimal model (StepCox + GBM). The ensemble framework was implemented using the ‘Mime1’ R package (version 0.13) to ensure reproducibility and systematic algorithm integration. For external validation cohorts, expression matrices were aligned to the training gene list, with missing genes imputed as zero to maintain feature consistency. Risk scores for each sample were predicted using the trained model with the optimal number of iterations, and samples were stratified into high- and low-risk groups based on the median risk score. Survival differences between risk groups were evaluated using Kaplan–Meier curves and log-rank tests, with hazard ratios (HRs) and 95% confidence intervals calculated. Time-dependent predictive performance was assessed by receiver operating characteristic (ROC) analysis at 1-, 3-, and 5-year time points, with the AUC used to quantify prognostic accuracy across time points.

### 4.16. Statistical Analysis

Statistical analyses were performed using R software (version 4.4.3) and GraphPad Prism (version 10.1.2). For continuous variables, differences were evaluated using the two-tailed Student’s t-test for normally distributed data, and the Wilcoxon rank-sum test or Kruskal–Wallis test for non-normally distributed data. Specifically, to compare single-cell feature scores (e.g., SAG score, CNV score, pseudotime values, and the expression levels of *MIF*, *APP*, *CD74*, *CD44*, and *CXCR4*) across subpopulations, the Kruskal–Wallis test was employed to evaluate global significance, followed by Wilcoxon rank-sum tests to compare individual cell clusters against the pooled global baseline. Furthermore, categorical differences and correlations between variables were evaluated using the chi-square test and Spearman’s rank correlation analysis, respectively. *p*-values derived from multiple comparisons were adjusted using the Benjamini–Hochberg procedure to control the false discovery rate (FDR). Statistical significance was defined as follows: * *p* < 0.05, ** *p* < 0.01, *** *p* < 0.001, **** *p* < 0.0001, and ns (not significant).

## 5. Conclusions

This study elucidates how the TME accelerates myeloid senescence. By establishing MSAG as a novel framework for immunosenescence, we provide a pan-cancer prognostic model with significant clinical translation potential.

## Data Availability

The original contributions presented in this study are included in the article. Further inquiries can be directed to the corresponding authors.

## Supplementary Materials

The following supporting information can be downloaded at: https://www.mdpi.com/article/10.3390/ijms27135688/s1.

## Abbreviations

The following abbreviations are used in this manuscript:

TME	Tumor Microenvironment
MSAG	Myeloid Senescence-associated Gene
DAMPs	Damage-associated Molecular Patterns
SASP	Senescence-associated Secretory Phenotype
GTEx	Genotype-Tissue Expression
SAGs	Senescence-associated Genes
CNV	Copy Number Variations
ccRCC	Clear Cell Renal Cell Carcinoma
GC	Gastric Carcinoma
NSCLC	Non-small-cell Lung Cancer
OV	Ovarian Cancer
DCs	Dendritic Cells
Mono/Macro	Monocytes/Macrophages
PPI	Protein–Protein Interaction
CDF	Cumulative Distribution Function
RSF	Random Survival Forest
AUC	Area Under the Curve
GEO	Gene Expression Omnibus
GAG	Glycosaminoglycan
ssGSEA	Single-sample Gene Set Enrichment Analysis
BRCA	Breast Carcinoma
RCC	Renal Cell Carcinoma
CRC	Colorectal Cancer
HNSCC	Head and Neck Squamous Cell Carcinoma
PDAC	Pancreatic Ductal Adenocarcinoma
PRAD	Prostate Adenocarcinoma
PCA	Principal Component Analysis
UMAP	Uniform Manifold Approximation and Projection
EBI	European Bioinformatics Institute
MNC	Maximum Neighborhood Component
CDS	Cell Data Set
TF	Transcription Factor
RSS	Regulon Specificity Scores
PAM	Partitioning Around Medoids
HRs	Hazard Ratios
ROC	Receiver Operating Characteristic
FDR	False Discovery Rate
