# Supplementary material for: Tumor Microenvironment Hijacks and Accelerates a Physiological Myeloid Senescence Signature Associated with Pan-Cancer Immunosuppression and Prognostic Stratification
Source: Int J Mol Sci. 2026 Jun 24;27(13):5688. doi: 10.3390/ijms27135688 (PMC13361799; doi:10.3390/ijms27135688)
Supplement: Supplementary file 1 [file ijms-27-05688-s001.zip › ijms-4358368-Supplementary Materials.pdf]

SA

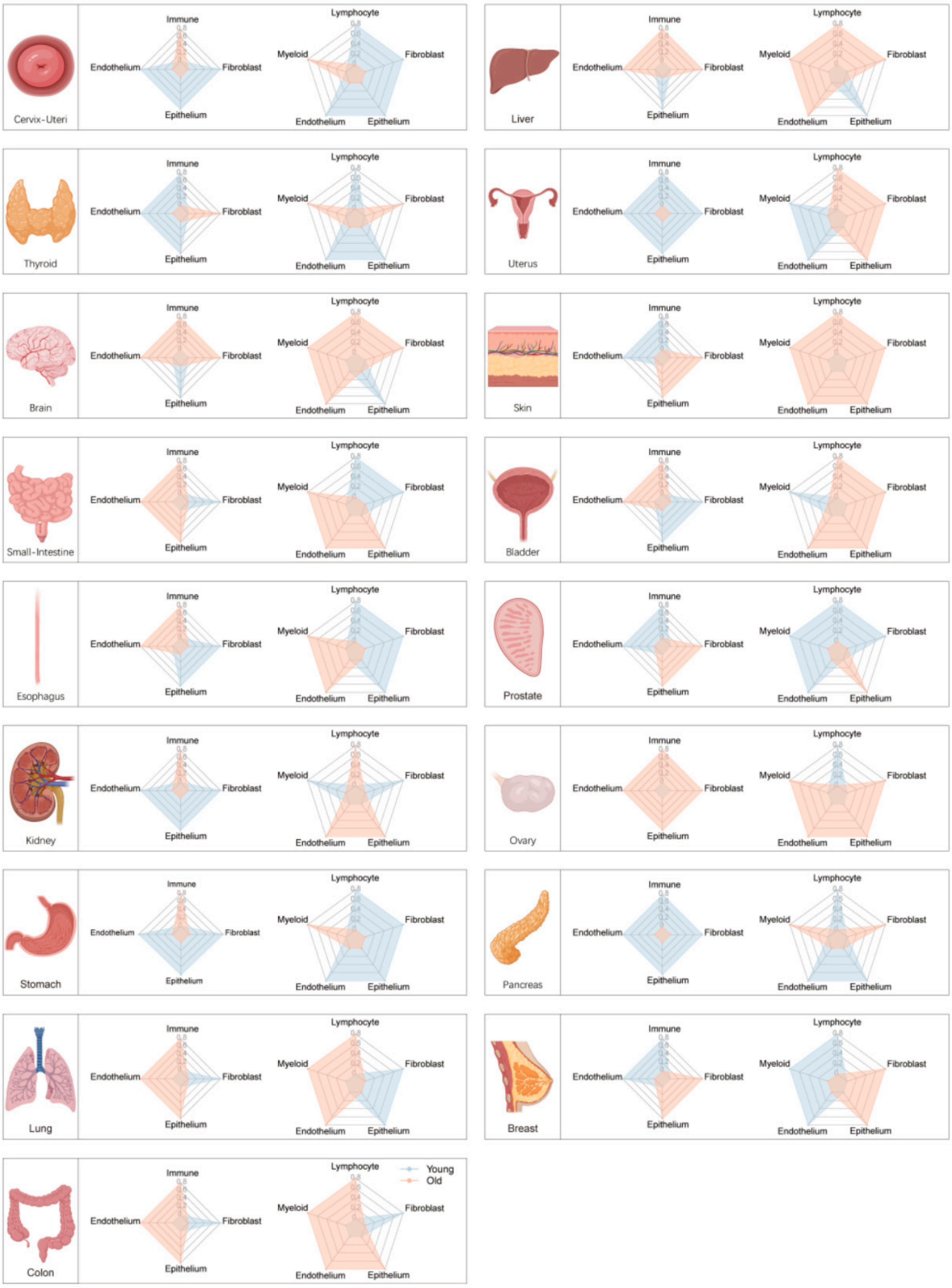

Cervix-Uteri
Immune
Endothelium
Fibroblast
Epithelium
Lymphocyte
Myeloid
Fibroblast
Endothelium
Epithelium
Liver
Immune
Endothelium
Fibroblast
Epithelium
Lymphocyte
Myeloid
Fibroblast
Endothelium
Epithelium
Thyroid
Immune
Endothelium
Fibroblast
Epithelium
Lymphocyte
Myeloid
Fibroblast
Endothelium
Epithelium
Uterus
Immune
Endothelium
Fibroblast
Epithelium
Lymphocyte
Myeloid
Fibroblast
Endothelium
Epithelium
Brain
Immune
Endothelium
Fibroblast
Epithelium
Lymphocyte
Myeloid
Fibroblast
Endothelium
Epithelium
Skin
Immune
Endothelium
Fibroblast
Epithelium
Lymphocyte
Myeloid
Fibroblast
Endothelium
Epithelium
Small-Intestine
Immune
Endothelium
Fibroblast
Epithelium
Lymphocyte
Myeloid
Fibroblast
Endothelium
Epithelium
Bladder
Immune
Endothelium
Fibroblast
Epithelium
Lymphocyte
Myeloid
Fibroblast
Endothelium
Epithelium
Esophagus
Immune
Endothelium
Fibroblast
Epithelium
Lymphocyte
Myeloid
Fibroblast
Endothelium
Epithelium
Prostate
Immune
Endothelium
Fibroblast
Epithelium
Lymphocyte
Myeloid
Fibroblast
Endothelium
Epithelium
Kidney
Immune
Endothelium
Fibroblast
Epithelium
Lymphocyte
Myeloid
Fibroblast
Endothelium
Epithelium
Ovary
Immune
Endothelium
Fibroblast
Epithelium
Lymphocyte
Myeloid
Fibroblast
Endothelium
Epithelium
Stomach
Immune
Endothelium
Fibroblast
Epithelium
Lymphocyte
Myeloid
Fibroblast
Endothelium
Epithelium
Pancreas
Immune
Endothelium
Fibroblast
Epithelium
Lymphocyte
Myeloid
Fibroblast
Endothelium
Epithelium
Lung
Immune
Endothelium
Fibroblast
Epithelium
Lymphocyte
Myeloid
Fibroblast
Endothelium
Epithelium
Breast
Immune
Endothelium
Fibroblast
Epithelium
Lymphocyte
Myeloid
Fibroblast
Endothelium
Epithelium
Colon
Immune
Endothelium
Fibroblast
Epithelium
Lymphocyte
Myeloid
Fibroblast
Endothelium
Epithelium
Young
Old

SB

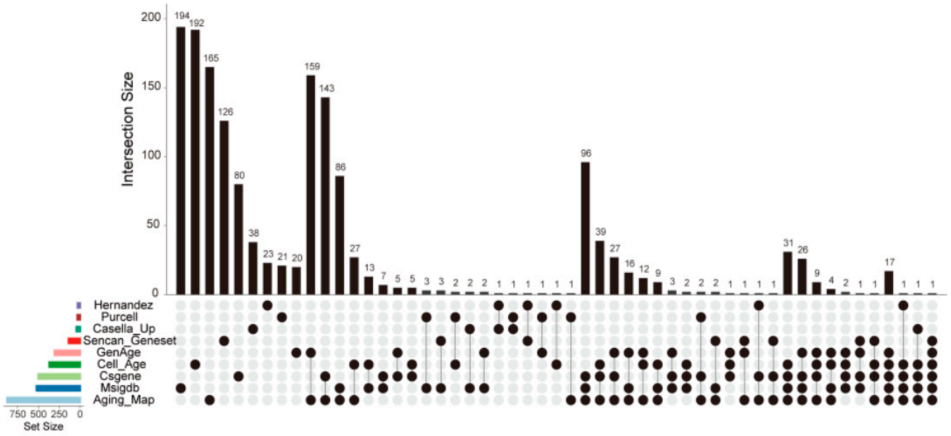
Intersection Size
200
150
100
50
0
194
192
165
126
80
38
23
21
20
159
143
86
27
13
7
5
5
3
3
2
2
2
1
1
1
1
1
1
96
39
27
16
12
9
3
2
2
2
1
1
1
1
31
26
9
4
2
1
1
17
1
1
1
Hernandez
Purcell
Casella_Up
Sencan_Geneset
GenAge
Cell_Age
Csgene
Msigdb
Aging_Map
750 500 250 0
Set Size

SC

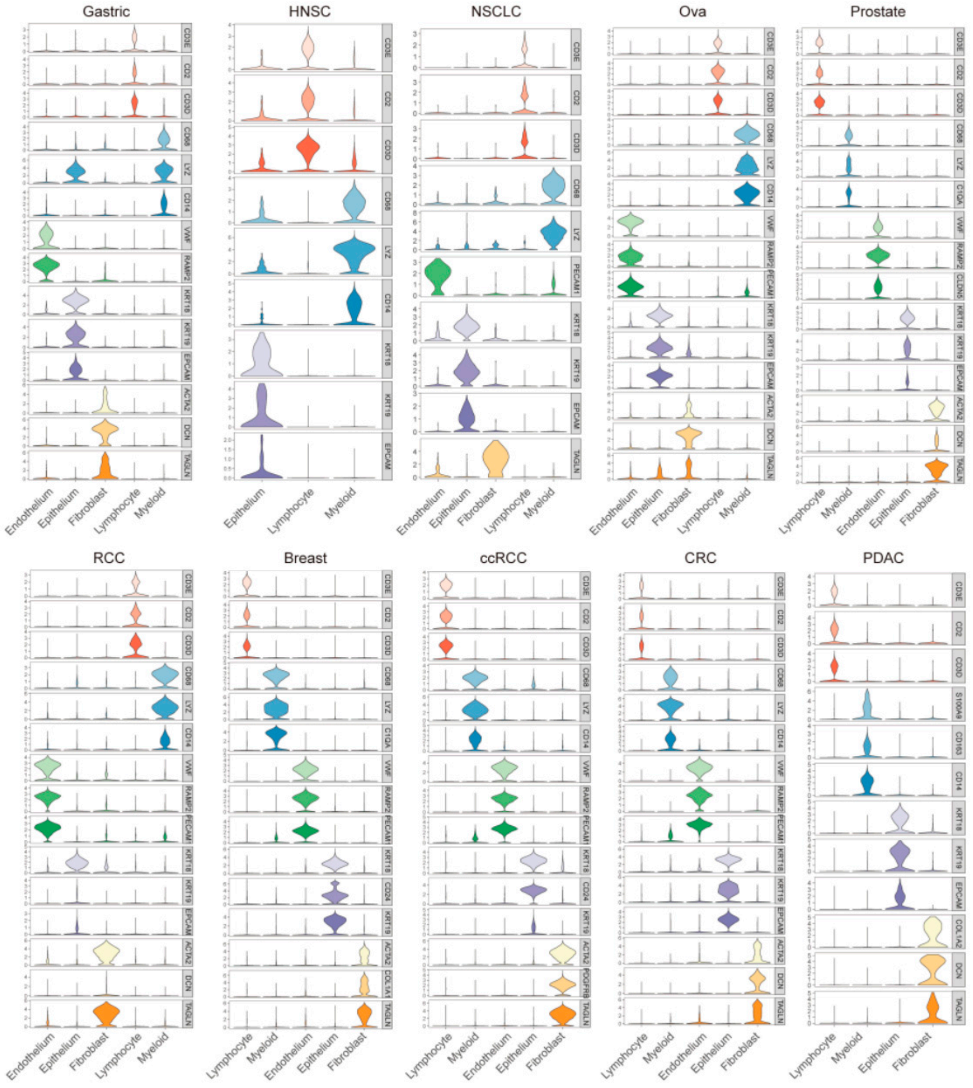
Gastric
HNSC
NSCLC
Ova
Prostate
Endothelium
Epithelium
Fibroblast
Lymphocyte
Myeloid
RCC
Breast
ccRCC
CRC
PDAC

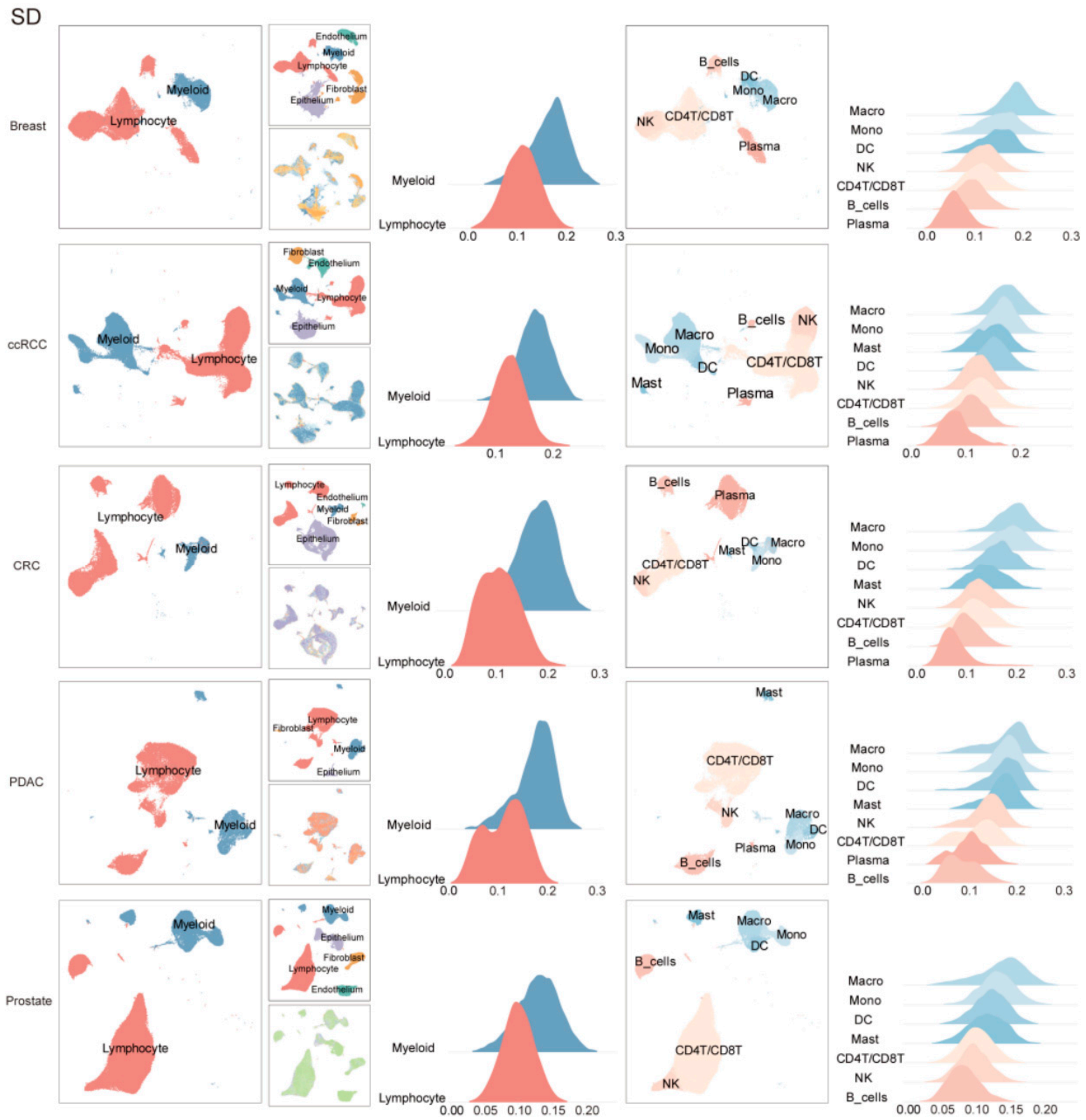

SD
Breast
ccRCC
CRC
PDAC
Prostate
Myeloid
Lymphocyte
Endothelium
Fibroblast
Epithelium
Macro
Mono
DC
Mast
NK
CD4T/CD8T
B_cells
Plasma

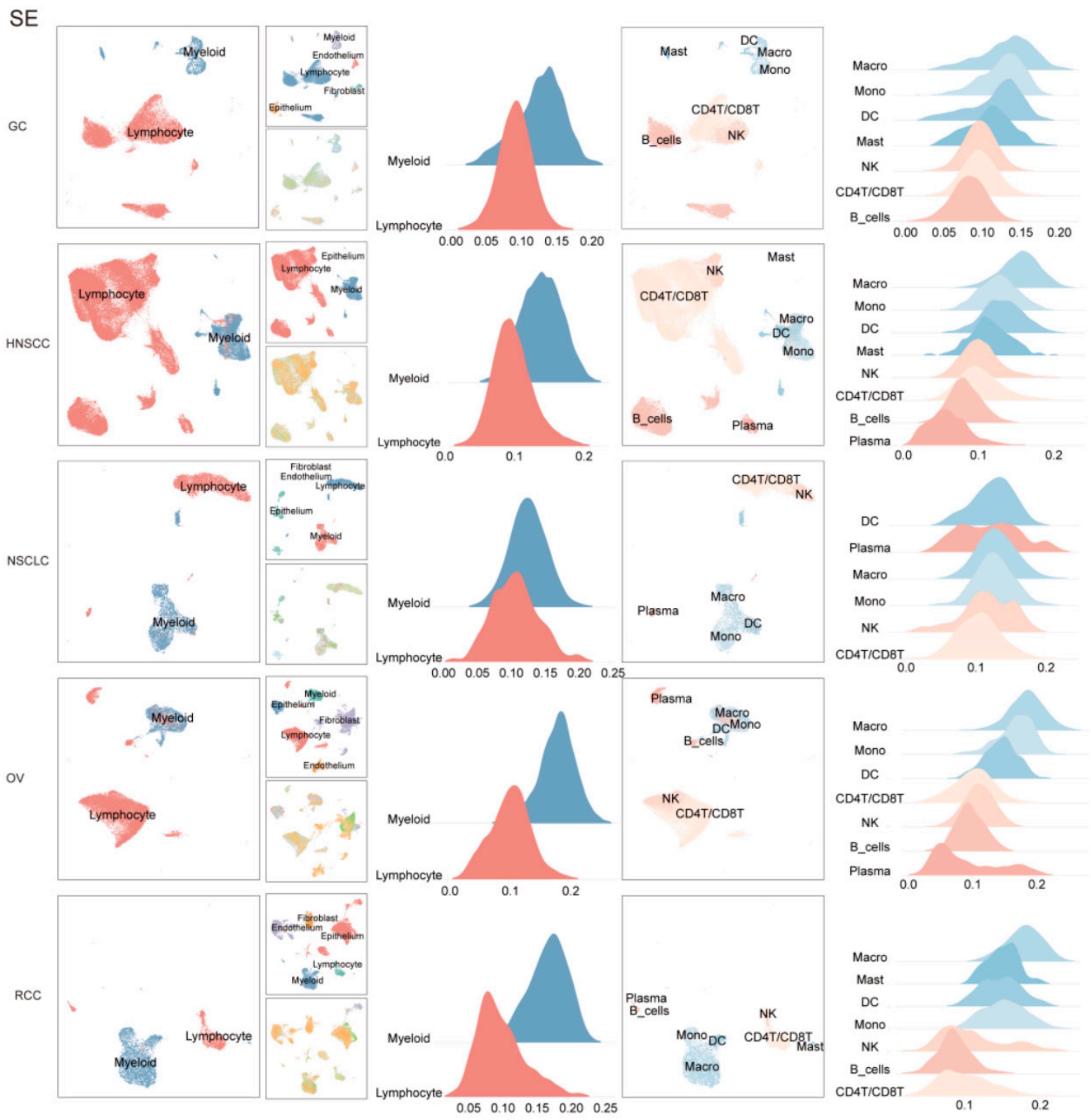

Supplementary Figure S1. The landscape of age-related cellular infiltration and immunosenescence across normal tissues and multiple cancer types. (**A**) Radar plots comparing cell infiltration patterns in normal tissues across different age groups. Organ schematics were created in BioRender. Jiang, H. (2026). (**B**) UpSet plot illustrating the selection of SAGs. (**C**) Violin plots of marker gene expression for cell types in pan-cancer single-cell datasets. Cells were classified into five major categories, including Myeloid, Lymphocyte, Fibroblast, Endothelium, and Epithelium, based on canonical markers. (**D–E**) Pan-cancer single-cell UMAP projections and ridge plots of senescence scores.

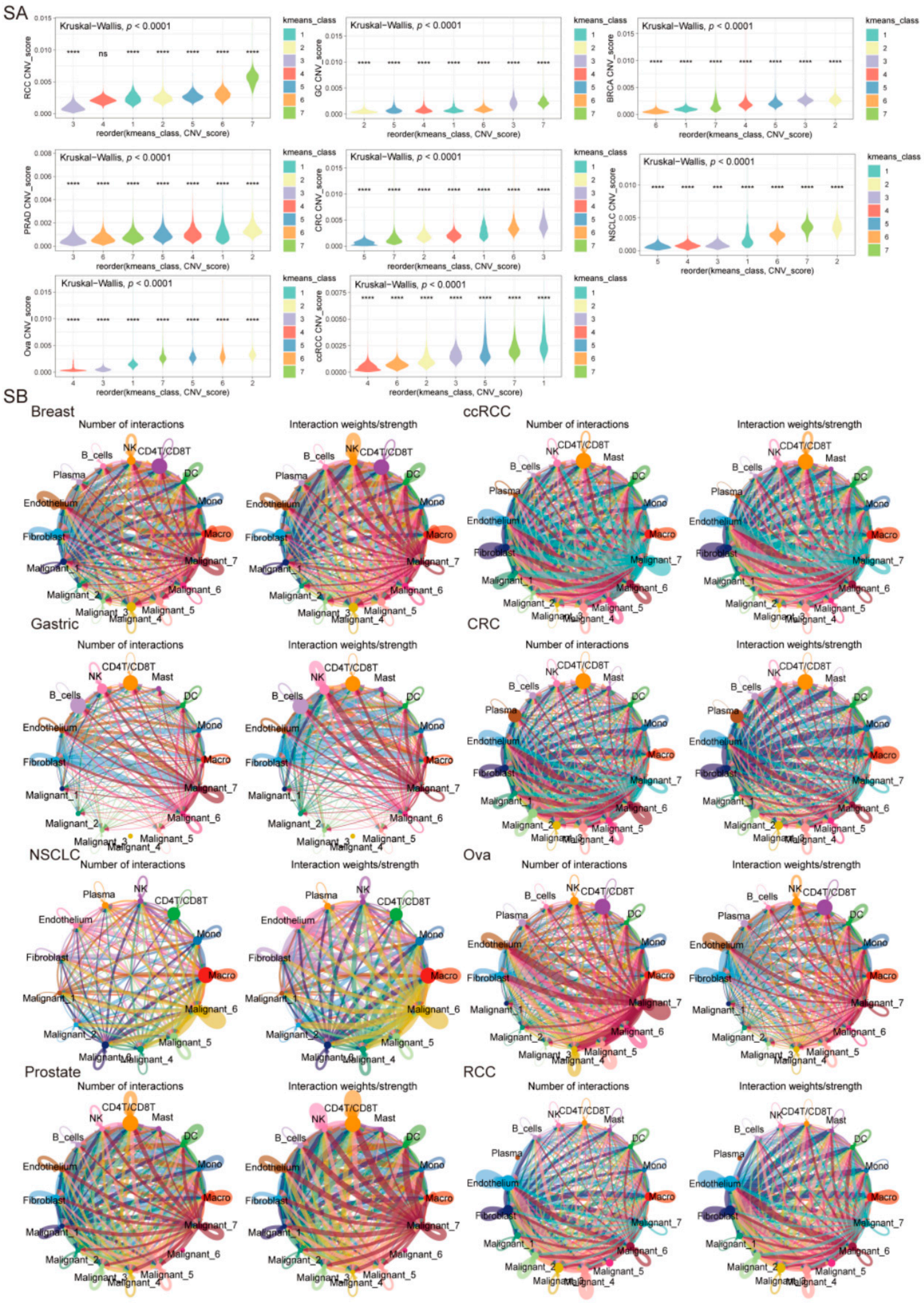
SA
Kruskal−Wallis, p < 0.0001
RCC CNV_score
GC CNV_score
BRCA CNV_score
PRAD CNV_score
CRC CNV_score
NSCLC CNV_score
Ova CNV_score
ccRCC CNV_score
reorder(kmeans_class, CNV_score)
kmeans_class
SB
Breast
ccRCC
Gastric
CRC
NSCLC
Ova
Prostate
RCC
Number of interactions
Interaction weights/strength
B_cells
NK
CD4T/CD8T
Mast
DC
Mono
Macro
Plasma
Endothelium
Fibroblast
Malignant_1
Malignant_2
Malignant_3
Malignant_4
Malignant_5
Malignant_6
Malignant_7

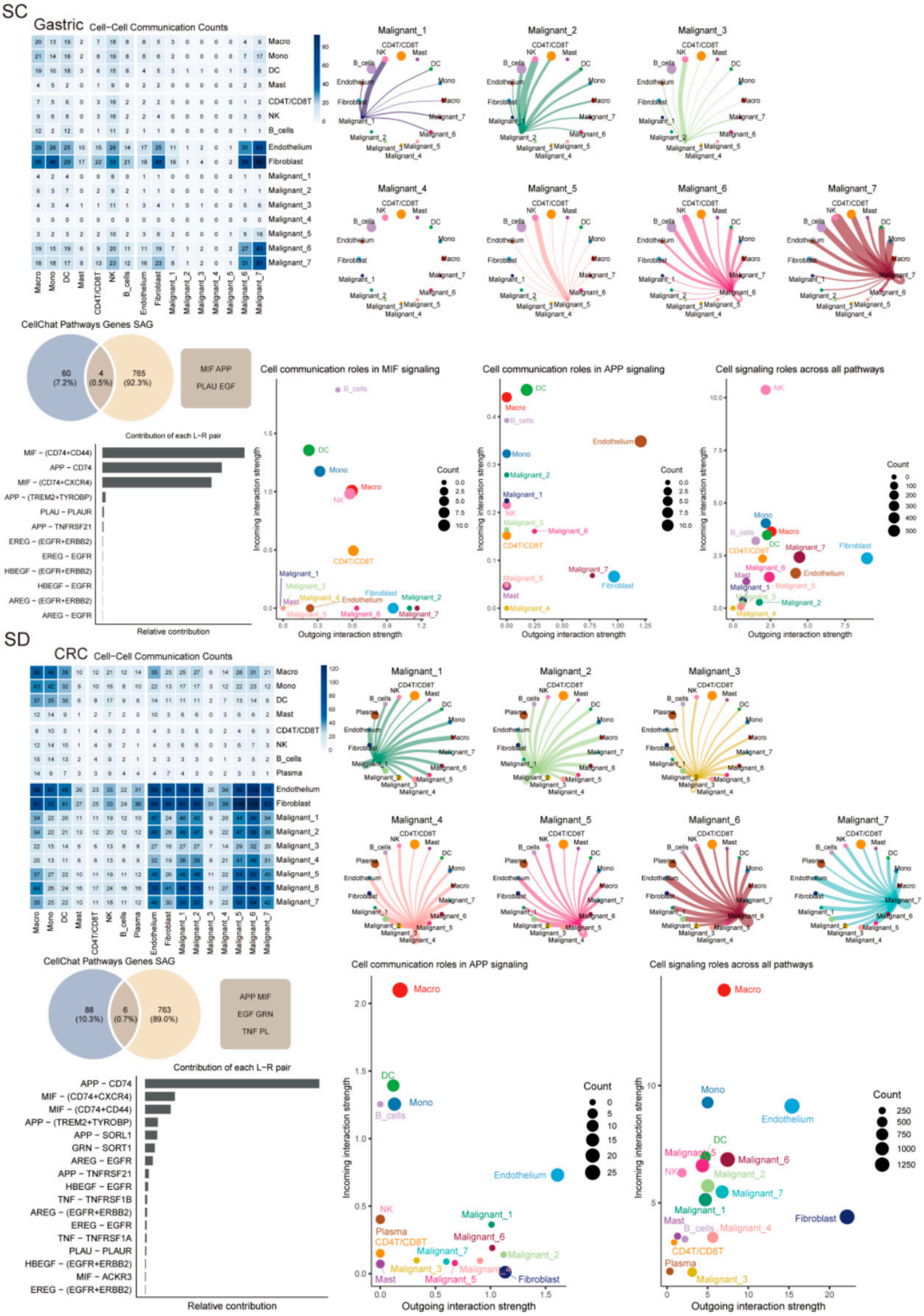
SC
Gastric
Cell-Cell Communication Counts
Malignant_1
Malignant_2
Malignant_3
Malignant_4
Malignant_5
Malignant_6
Malignant_7
CellChat Pathways Genes SAG
60 (7.2%)
4 (0.5%)
765 (92.3%)
MIF APP
PLAU EGF
Contribution of each L-R pair
MIF - (CD74+CD44)
APP - CD74
MIF - (CD74+CXCR4)
APP - (TREM2+TYROBP)
PLAU - PLAUR
APP - TNFRSF21
EREG - (EGFR+ERBB2)
EREG - EGFR
HBEGF - (EGFR+ERBB2)
HBEGF - EGFR
AREG - (EGFR+ERBB2)
AREG - EGFR
Relative contribution
Cell communication roles in MIF signaling
Cell communication roles in APP signaling
Cell signaling roles across all pathways
Incoming interaction strength
Outgoing interaction strength
Count
SD
CRC
Cell-Cell Communication Counts
CellChat Pathways Genes SAG
88 (10.3%)
6 (0.7%)
763 (89.0%)
APP MIF
EGF GRN
TNF PL
Contribution of each L-R pair
APP - CD74
MIF - (CD74+CXCR4)
MIF - (CD74+CD44)
APP - (TREM2+TYROBP)
APP - SORL1
GRN - SORT1
AREG - EGFR
APP - TNFRSF21
HBEGF - EGFR
TNF - TNFRSF1B
AREG - (EGFR+ERBB2)
EREG - EGFR
TNF - TNFRSF1A
PLAU - PLAUR
HBEGF - (EGFR+ERBB2)
MIF - ACKR3
EREG - (EGFR+ERBB2)
Relative contribution
Cell communication roles in APP signaling
Cell signaling roles across all pathways
Incoming interaction strength
Outgoing interaction strength
Count

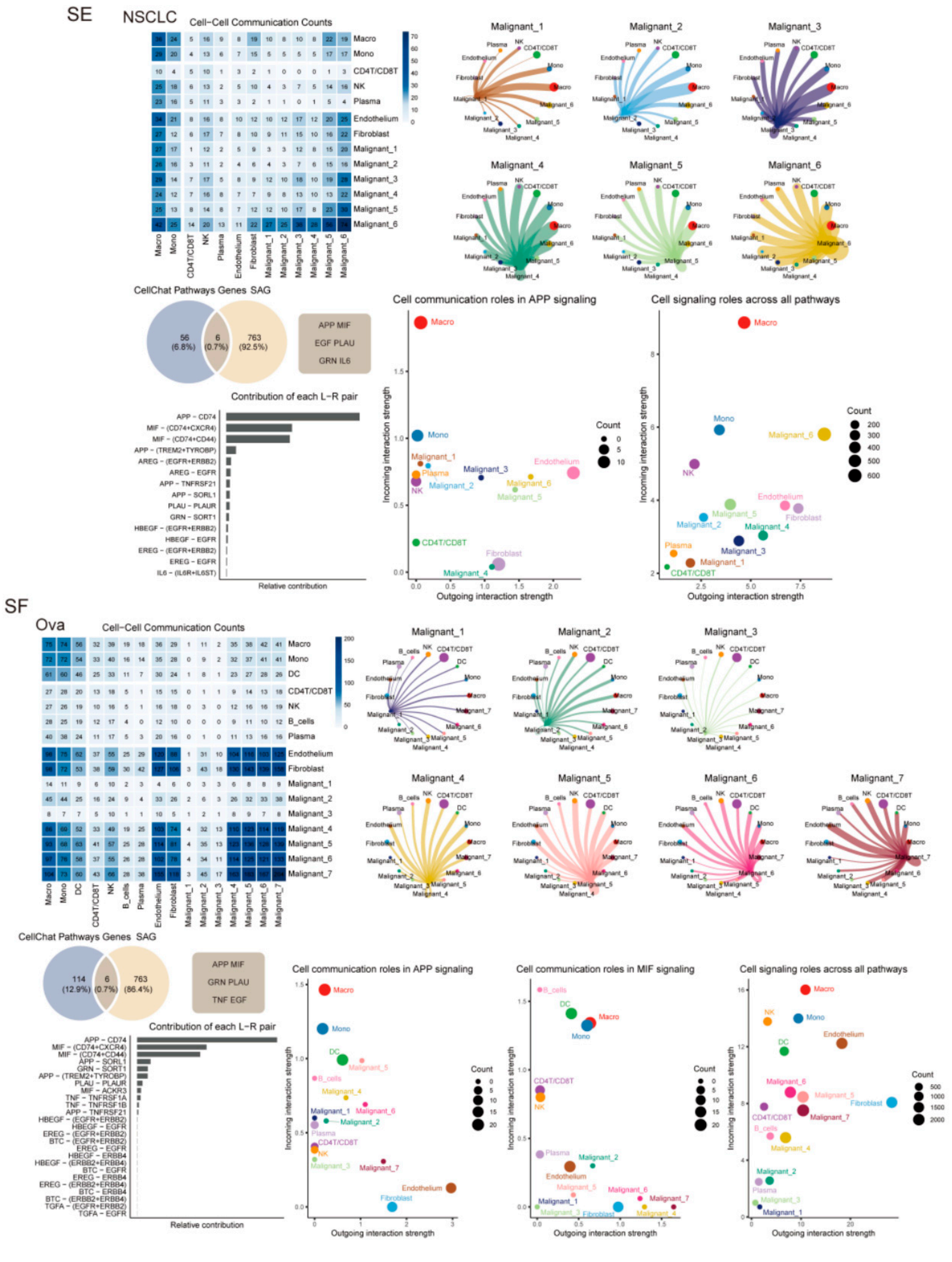

SE
NSCLC
Cell-Cell Communication Counts
Malignant_1
Malignant_2
Malignant_3
Malignant_4
Malignant_5
Malignant_6
CellChat Pathways Genes SAG
56 (6.8%)
6 (0.7%)
763 (92.5%)
APP MIF
EGF PLAU
GRN IL6
Contribution of each L-R pair
Relative contribution
Cell communication roles in APP signaling
Cell signaling roles across all pathways
Incoming interaction strength
Outgoing interaction strength
Count
SF
Ova
Cell-Cell Communication Counts
Malignant_7
CellChat Pathways Genes SAG
114 (12.9%)
6 (0.7%)
763 (86.4%)
APP MIF
GRN PLAU
TNF EGF
Contribution of each L-R pair
Relative contribution
Cell communication roles in APP signaling
Cell communication roles in MIF signaling
Cell signaling roles across all pathways

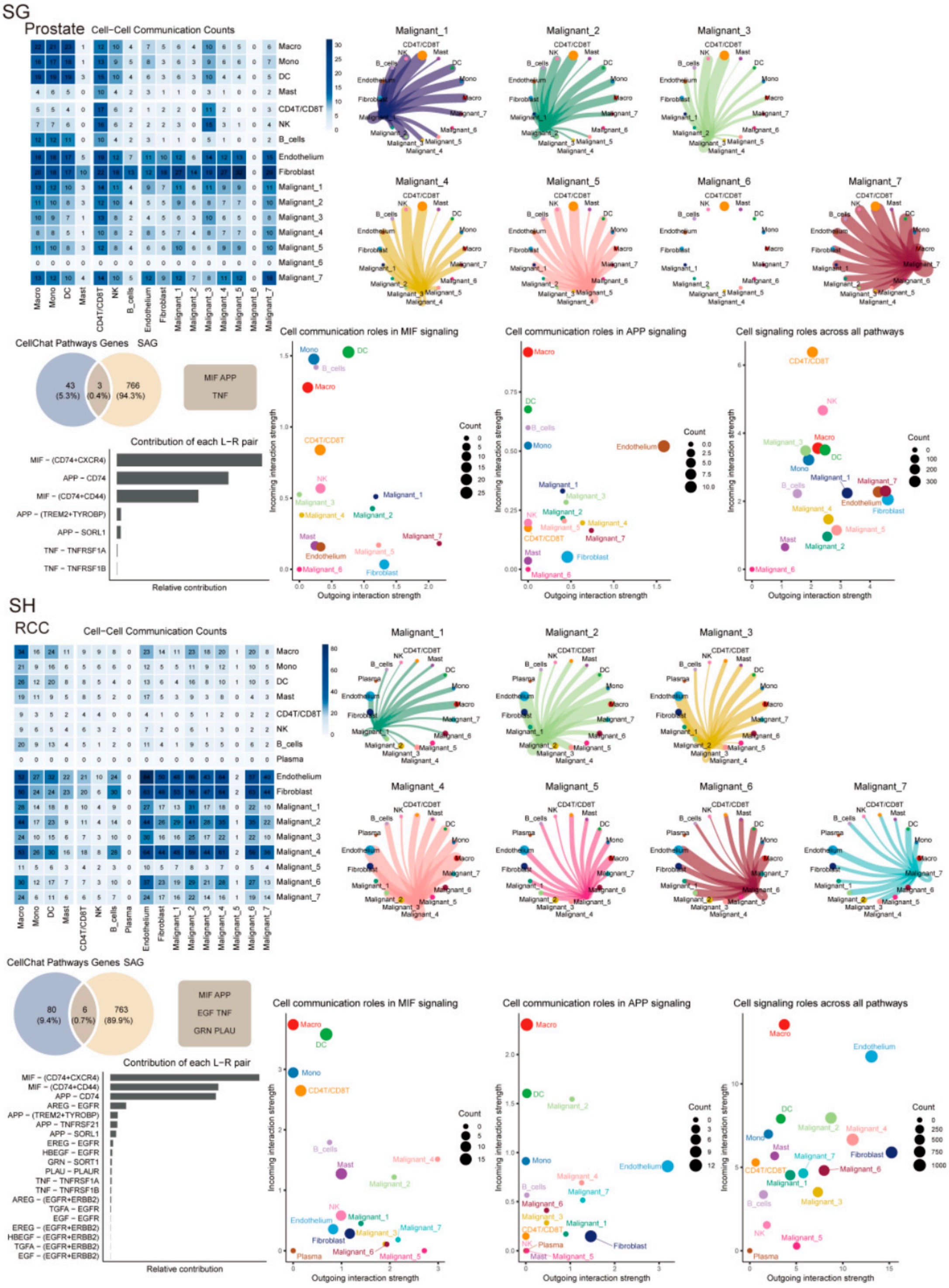
SG
Prostate
Cell-Cell Communication Counts
Malignant_1
Malignant_2
Malignant_3
Malignant_4
Malignant_5
Malignant_6
Malignant_7
CellChat Pathways Genes
SAG
MIF APP
TNF
Contribution of each L-R pair
MIF - (CD74+CXCR4)
APP - CD74
MIF - (CD74+CD44)
APP - (TREM2+TYROBP)
APP - SORL1
TNF - TNFRSF1A
TNF - TNFRSF1B
Relative contribution
Cell communication roles in MIF signaling
Cell communication roles in APP signaling
Cell signaling roles across all pathways
Incoming interaction strength
Outgoing interaction strength
Count
SH
RCC
Cell-Cell Communication Counts
CellChat Pathways Genes SAG
MIF APP
EGF TNF
GRN PLAU
Contribution of each L-R pair
MIF - (CD74+CXCR4)
MIF - (CD74+CD44)
APP - CD74
AREG - EGFR
APP - (TREM2+TYROBP)
APP - TNFRSF21
APP - SORL1
EREG - EGFR
HBEGF - EGFR
GRN - SORT1
PLAU - PLAUR
TNF - TNFRSF1A
TNF - TNFRSF1B
AREG - (EGFR+ERBB2)
TGFA - EGFR
EGF - EGFR
EREG - (EGFR+ERBB2)
HBEGF - (EGFR+ERBB2)
TGFA - (EGFR+ERBB2)
EGF - (EGFR+ERBB2)
Relative contribution
Cell communication roles in MIF signaling
Cell communication roles in APP signaling
Cell signaling roles across all pathways

SI

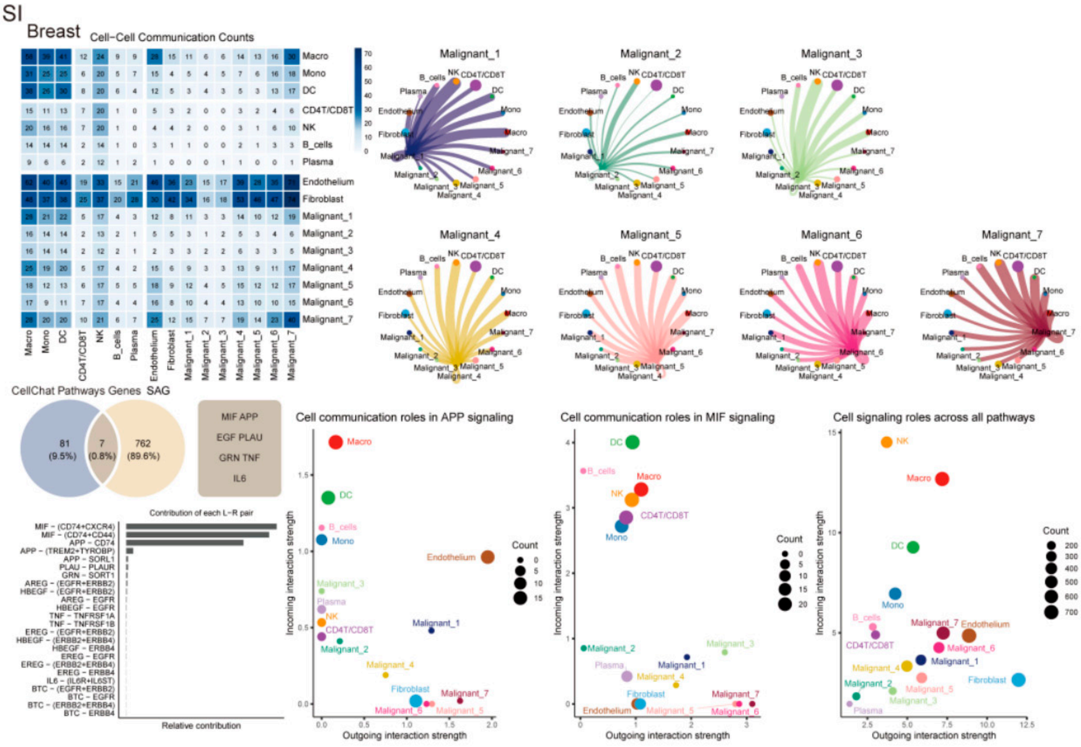
Breast
Cell-Cell Communication Counts
CellChat Pathways Genes
SAG
MIF APP
EGF PLAU
GRN TNF
IL6
Contribution of each L-R pair
Relative contribution
Cell communication roles in APP signaling
Cell communication roles in MIF signaling
Cell signaling roles across all pathways
Outgoing interaction strength
Incoming interaction strength

SJ

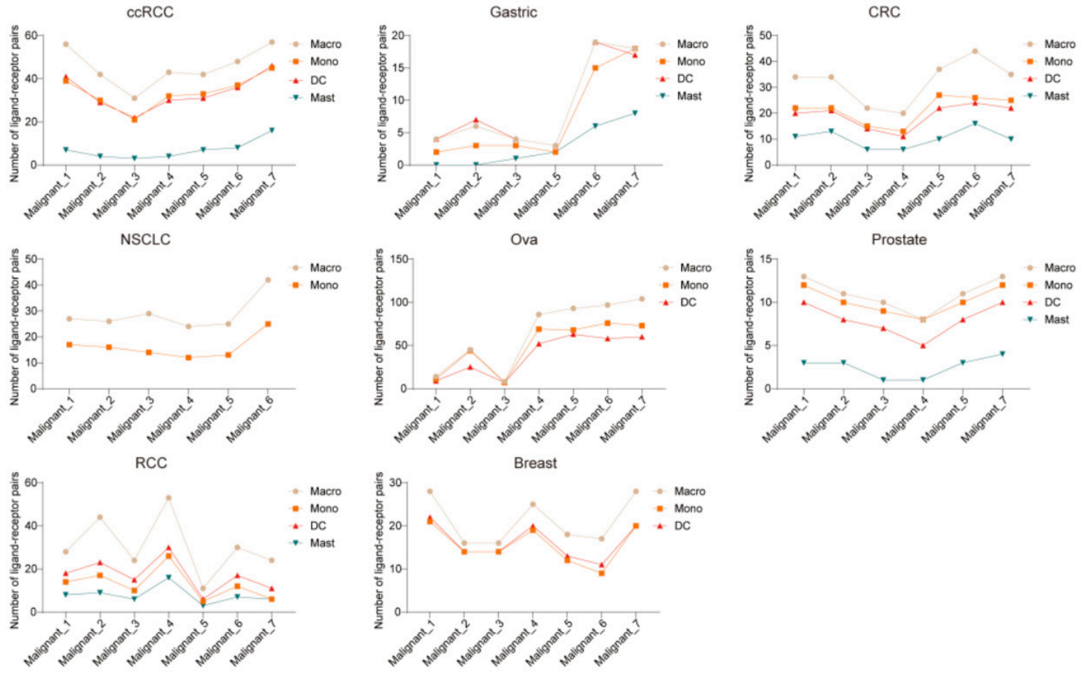
ccRCC
Gastric
CRC
NSCLC
Ova
Prostate
RCC
Breast
Number of ligand-receptor pairs
Macro
Mono
DC
Mast

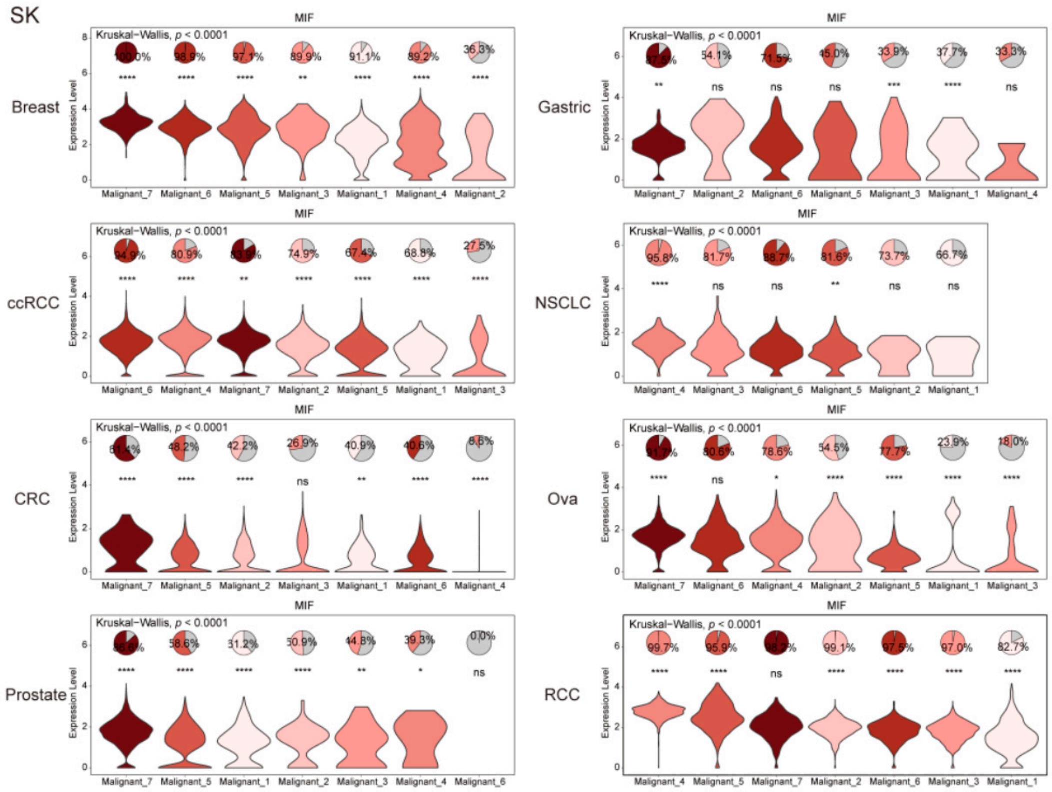
SK
Breast
Gastric
ccRCC
NSCLC
CRC
Ova
Prostate
RCC
MIF
Kruskal−Wallis, p < 0.0001
Expression Level
Malignant_1
Malignant_2
Malignant_3
Malignant_4
Malignant_5
Malignant_6
Malignant_7

SL

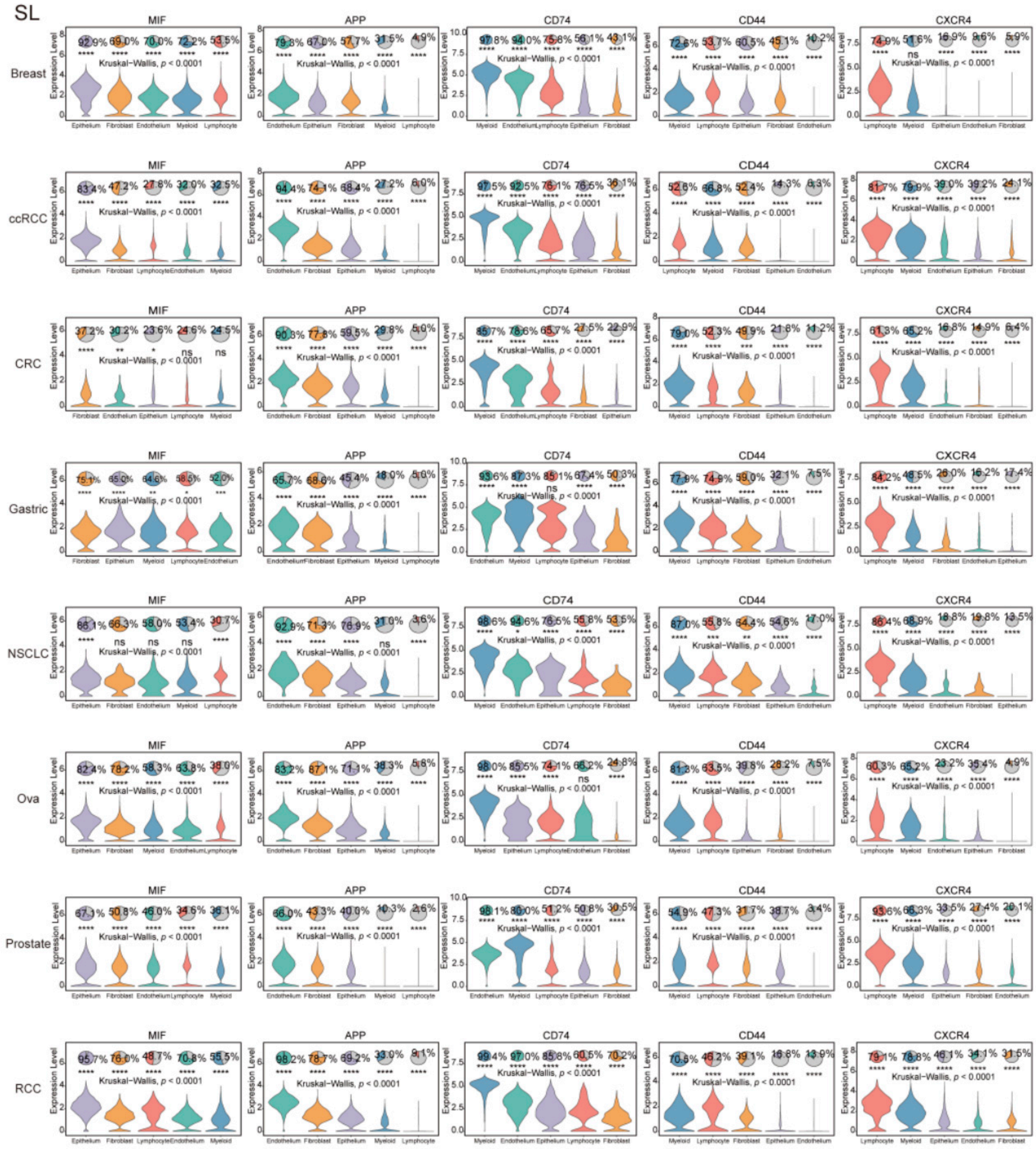
MIF
APP
CD74
CD44
CXCR4
Breast
ccRCC
CRC
Gastric
NSCLC
Ova
Prostate
RCC
Expression Level
Kruskal-Wallis, p < 0.0001
Epithelium
Fibroblast
Endothelium
Myeloid
Lymphocyte

SM

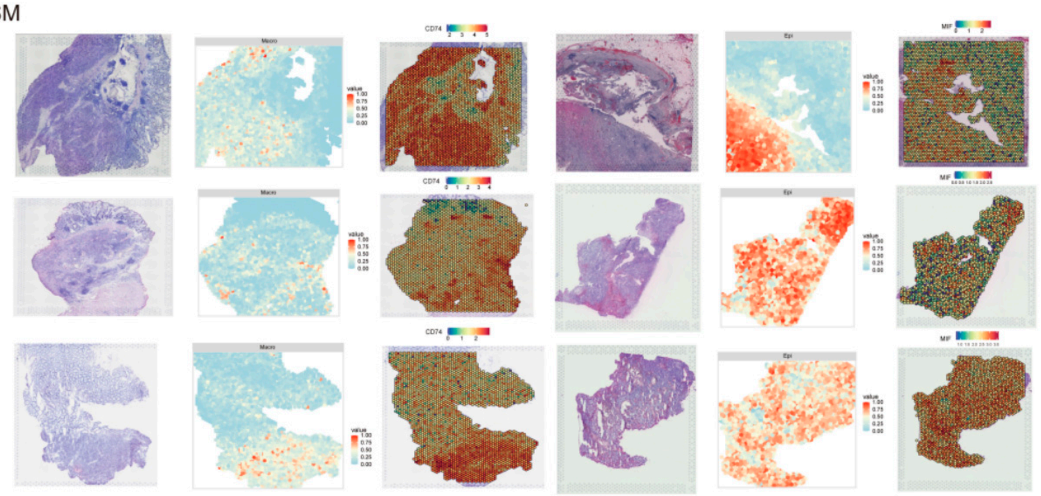
Macro
CD74
Epi
MIF
value

Supplementary Figure S2. Analysis of senescence-associated pathways mediating interactions between malignant epithelial cells and immune cells across cancers. (**A**) Pan-cancer epithelial cells clustered into seven subgroups based on CNV scores. (**B**) Number and strength of interactions among different cell types across cancers. (**C–I**) Quantitative analyses of interaction number and strength between malignant epithelial cells and immune cells across cancers, with identifying key senescence-associated pathway genes. Each panel depicts signaling pathways specific to each cancer type and the associated cell populations that emit or receive senescence signals. (**J**) Changes in ligand-receptor interaction numbers between epithelial subclusters of varying malignancy and myeloid subclusters. (**K**) Expression and distribution of *MIF* in pan-cancer malignant epithelial subclusters. (**L**) Expression and distribution of genes involved in senescence-related ligand-receptor pairs (*MIF-CD74+CXCR4* and *MIF-CD74+CD44*) across pan-cancer cell types. (**M**) Spatial expression abundance of *CD74* and *MIF* and the corresponding proportional abundance distributions of cell types (Macro and Epi) in representative tissue sections (including H&E staining). The statistical difference was analyzed by Wilcoxon rank-sum tests with Benjamini-Hochberg adjustment, where **** represents adjusted $P < 0.0001$, *** represents adjusted $P < 0.001$, ** represents adjusted $P < 0.01$, * represents adjusted $P < 0.05$, and ns represents not significant.

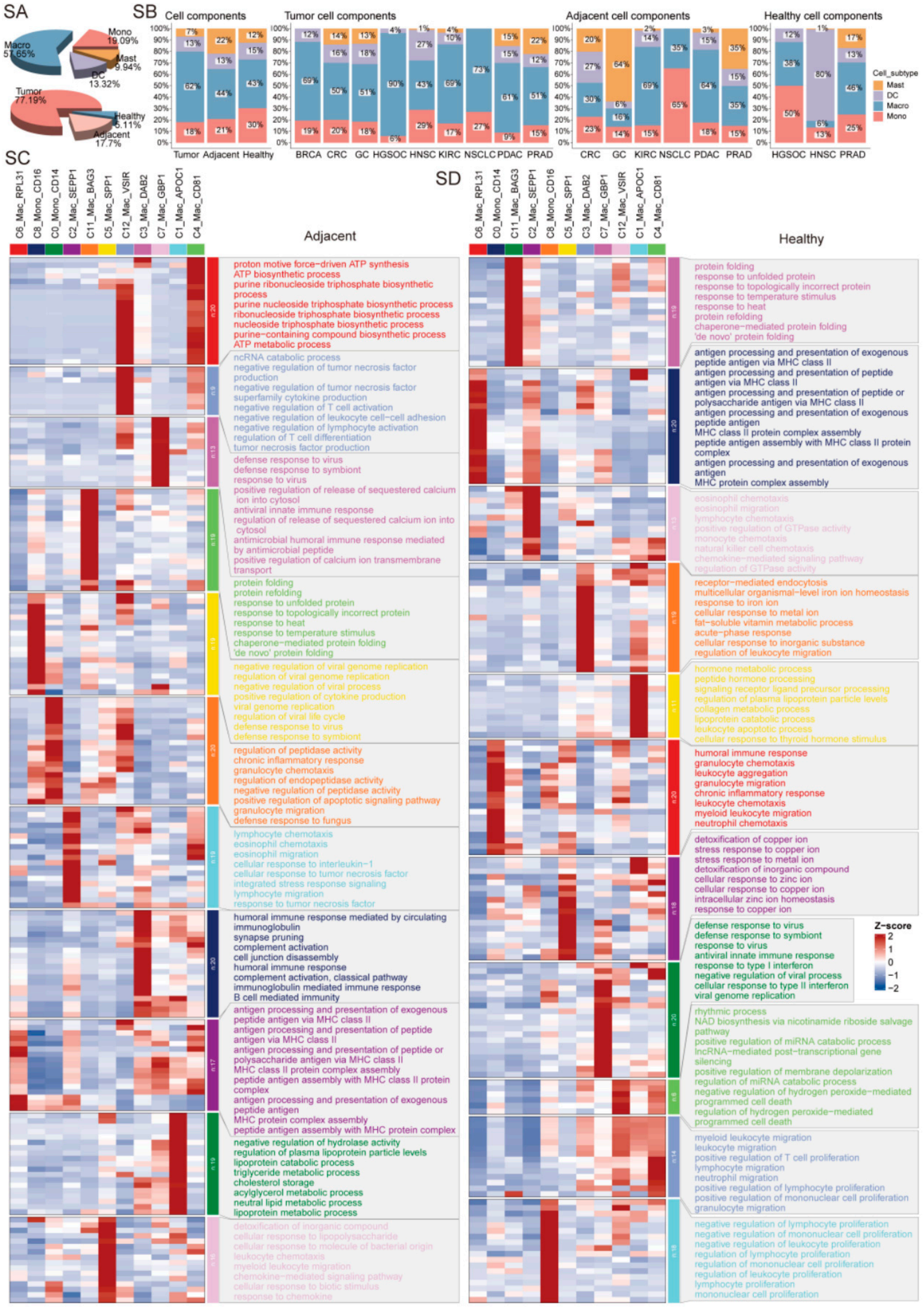

SA
Macro 57.65%
Mono 19.09%
Mast 9.94%
DC 13.32%
Tumor 77.19%
Healthy 5.11%
Adjacent 17.7%
SB
Cell components
Tumor cell components
Adjacent cell components
Healthy cell components
Tumor Adjacent Healthy
BRCA CRC GC HGSOC HNSC KIRC NSCLC PDAC PRAD
CRC GC KIRC NSCLC PDAC PRAD
HGSOC HNSC PRAD
Cell_subtype
Mast
DC
Macro
Mono
SC
C6_Mac_RPL31
C8_Mono_CD16
C0_Mono_CD14
C2_Mac_SEPP1
C11_Mac_BAG3
C5_Mac_SPP1
C12_Mac_VSIR
C3_Mac_DAB2
C7_Mac_GBP1
C1_Mac_APOC1
C4_Mac_CD81
Adjacent
proton motive force−driven ATP synthesis
ATP biosynthetic process
purine ribonucleoside triphosphate biosynthetic process
purine nucleoside triphosphate biosynthetic process
ribonucleoside triphosphate biosynthetic process
nucleoside triphosphate biosynthetic process
purine−containing compound biosynthetic process
ATP metabolic process
ncRNA catabolic process
negative regulation of tumor necrosis factor production
negative regulation of tumor necrosis factor superfamily cytokine production
negative regulation of T cell activation
negative regulation of leukocyte cell−cell adhesion
negative regulation of lymphocyte activation
regulation of T cell differentiation
tumor necrosis factor production
defense response to virus
defense response to symbiont
response to virus
positive regulation of release of sequestered calcium ion into cytosol
antiviral innate immune response
regulation of release of sequestered calcium ion into cytosol
antimicrobial humoral immune response mediated by antimicrobial peptide
positive regulation of calcium ion transmembrane transport
protein folding
protein refolding
response to unfolded protein
response to topologically incorrect protein
response to heat
response to temperature stimulus
chaperone−mediated protein folding
'de novo' protein folding
negative regulation of viral genome replication
regulation of viral genome replication
negative regulation of viral process
positive regulation of cytokine production
viral genome replication
regulation of viral life cycle
defense response to virus
defense response to symbiont
regulation of peptidase activity
chronic inflammatory response
granulocyte chemotaxis
regulation of endopeptidase activity
negative regulation of peptidase activity
positive regulation of apoptotic signaling pathway
granulocyte migration
defense response to fungus
lymphocyte chemotaxis
eosinophil chemotaxis
eosinophil migration
cellular response to interleukin−1
cellular response to tumor necrosis factor
integrated stress response signaling
lymphocyte migration
response to tumor necrosis factor
humoral immune response mediated by circulating immunoglobulin
synapse pruning
complement activation
cell junction disassembly
humoral immune response
complement activation, classical pathway
immunoglobulin mediated immune response
B cell mediated immunity
antigen processing and presentation of exogenous peptide antigen via MHC class II
antigen processing and presentation of peptide antigen via MHC class II
antigen processing and presentation of peptide or polysaccharide antigen via MHC class II
MHC class II protein complex assembly
peptide antigen assembly with MHC class II protein complex
antigen processing and presentation of exogenous peptide antigen
MHC protein complex assembly
peptide antigen assembly with MHC protein complex
negative regulation of hydrolase activity
regulation of plasma lipoprotein particle levels
lipoprotein catabolic process
triglyceride metabolic process
cholesterol storage
acylglycerol metabolic process
neutral lipid metabolic process
lipoprotein metabolic process
detoxification of inorganic compound
cellular response to lipopolysaccharide
cellular response to molecule of bacterial origin
leukocyte chemotaxis
myeloid leukocyte migration
chemokine−mediated signaling pathway
cellular response to biotic stimulus
response to chemokine
SD
C6_Mac_RPL31
C0_Mono_CD14
C11_Mac_BAG3
C2_Mac_SEPP1
C8_Mono_CD16
C5_Mac_SPP1
C3_Mac_DAB2
C7_Mac_GBP1
C12_Mac_VSIR
C1_Mac_APOC1
C4_Mac_CD81
Healthy
protein folding
response to unfolded protein
response to topologically incorrect protein
response to temperature stimulus
response to heat
protein refolding
chaperone−mediated protein folding
'de novo' protein folding
antigen processing and presentation of exogenous peptide antigen via MHC class II
antigen processing and presentation of peptide antigen via MHC class II
antigen processing and presentation of peptide or polysaccharide antigen via MHC class II
antigen processing and presentation of exogenous peptide antigen
MHC class II protein complex assembly
peptide antigen assembly with MHC class II protein complex
antigen processing and presentation of exogenous antigen
MHC protein complex assembly
eosinophil chemotaxis
eosinophil migration
lymphocyte chemotaxis
positive regulation of GTPase activity
monocyte chemotaxis
natural killer cell chemotaxis
chemokine−mediated signaling pathway
regulation of GTPase activity
receptor−mediated endocytosis
multicellular organismal−level iron ion homeostasis
response to iron ion
cellular response to metal ion
fat−soluble vitamin metabolic process
acute−phase response
cellular response to inorganic substance
regulation of leukocyte migration
hormone metabolic process
peptide hormone processing
signaling receptor ligand precursor processing
regulation of plasma lipoprotein particle levels
collagen metabolic process
lipoprotein catabolic process
leukocyte apoptotic process
cellular response to thyroid hormone stimulus
humoral immune response
granulocyte chemotaxis
leukocyte aggregation
granulocyte migration
chronic inflammatory response
leukocyte chemotaxis
myeloid leukocyte migration
neutrophil chemotaxis
detoxification of copper ion
stress response to copper ion
stress response to metal ion
detoxification of inorganic compound
cellular response to zinc ion
cellular response to copper ion
intracellular zinc ion homeostasis
response to copper ion
defense response to virus
defense response to symbiont
response to virus
antiviral innate immune response
response to type I interferon
negative regulation of viral process
cellular response to type II interferon
viral genome replication
Z−score
2
1
0
−1
−2
rhythmic process
NAD biosynthesis via nicotinamide riboside salvage pathway
positive regulation of miRNA catabolic process
lncRNA−mediated post−transcriptional gene silencing
positive regulation of membrane depolarization
regulation of miRNA catabolic process
negative regulation of hydrogen peroxide−mediated programmed cell death
regulation of hydrogen peroxide−mediated programmed cell death
myeloid leukocyte migration
leukocyte migration
positive regulation of T cell proliferation
lymphocyte migration
neutrophil migration
positive regulation of lymphocyte proliferation
positive regulation of mononuclear cell proliferation
granulocyte migration
negative regulation of lymphocyte proliferation
negative regulation of mononuclear cell proliferation
negative regulation of leukocyte proliferation
regulation of lymphocyte proliferation
regulation of mononuclear cell proliferation
regulation of leukocyte proliferation
lymphocyte proliferation
mononuclear cell proliferation

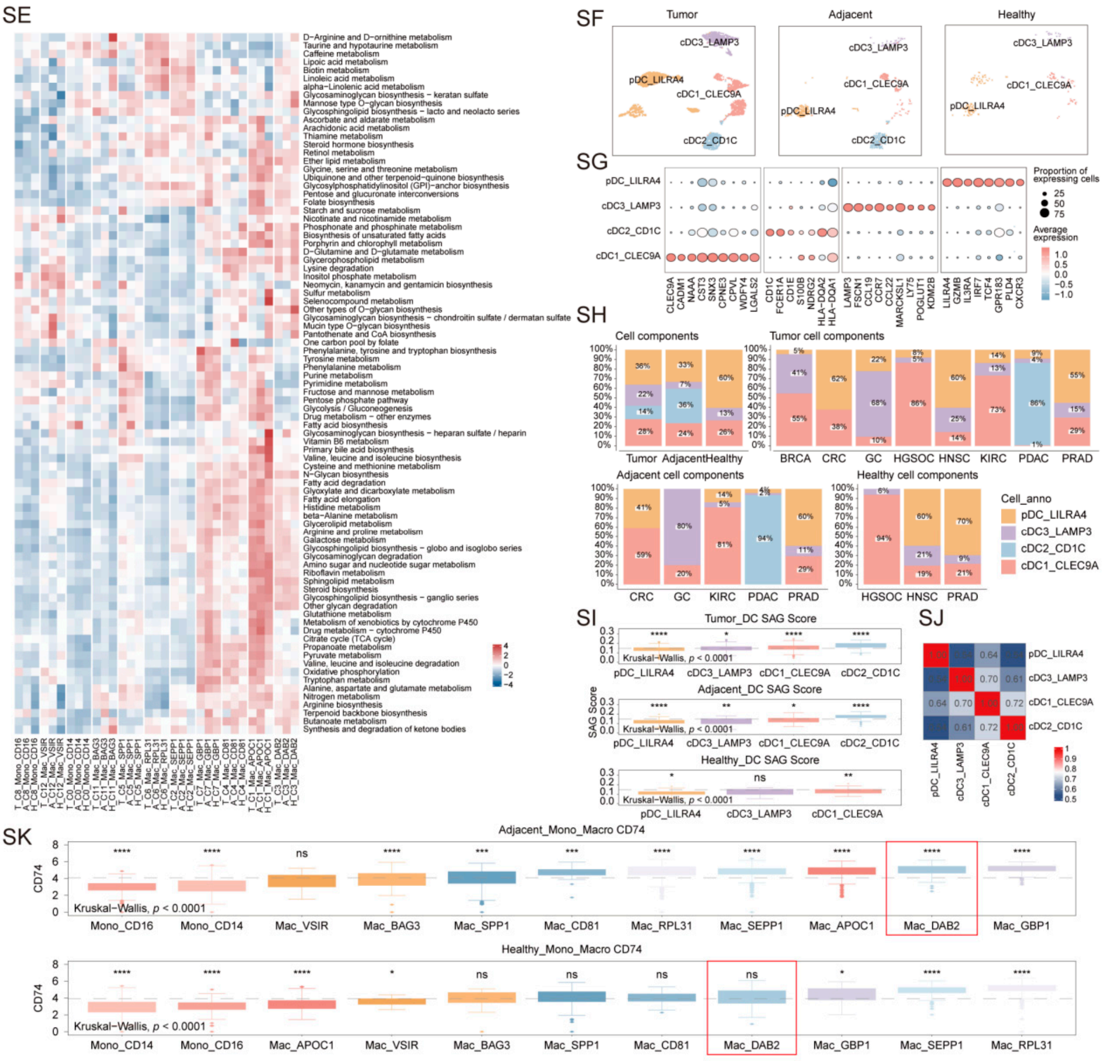

SE
SF
Tumor
Adjacent
Healthy
cDC3_LAMP3
pDC_LILRA4
cDC1_CLEC9A
cDC2_CD1C
SG
Proportion of expressing cells
25
50
75
Average expression
SH
Cell components
Tumor cell components
Tumor Adjacent Healthy
BRCA CRC GC HGSOC HNSC KIRC PDAC PRAD
Adjacent cell components
Healthy cell components
CRC GC KIRC PDAC PRAD
HGSOC HNSC PRAD
Cell_anno
pDC_LILRA4
cDC3_LAMP3
cDC2_CD1C
cDC1_CLEC9A
SI
Tumor_DC SAG Score
Adjacent_DC SAG Score
Healthy_DC SAG Score
SAG Score
Kruskal−Wallis, p < 0.0001
SJ
0.64
0.70
0.61
0.72
SK
Adjacent_Mono_Macro CD74
CD74
Mono_CD16 Mono_CD14 Mac_VSIR Mac_BAG3 Mac_SPP1 Mac_CD81 Mac_RPL31 Mac_SEPP1 Mac_APOC1 Mac_DAB2 Mac_GBP1
Healthy_Mono_Macro CD74
Mono_CD14 Mono_CD16 Mac_APOC1 Mac_VSIR Mac_BAG3 Mac_SPP1 Mac_CD81 Mac_DAB2 Mac_GBP1 Mac_SEPP1 Mac_RPL31

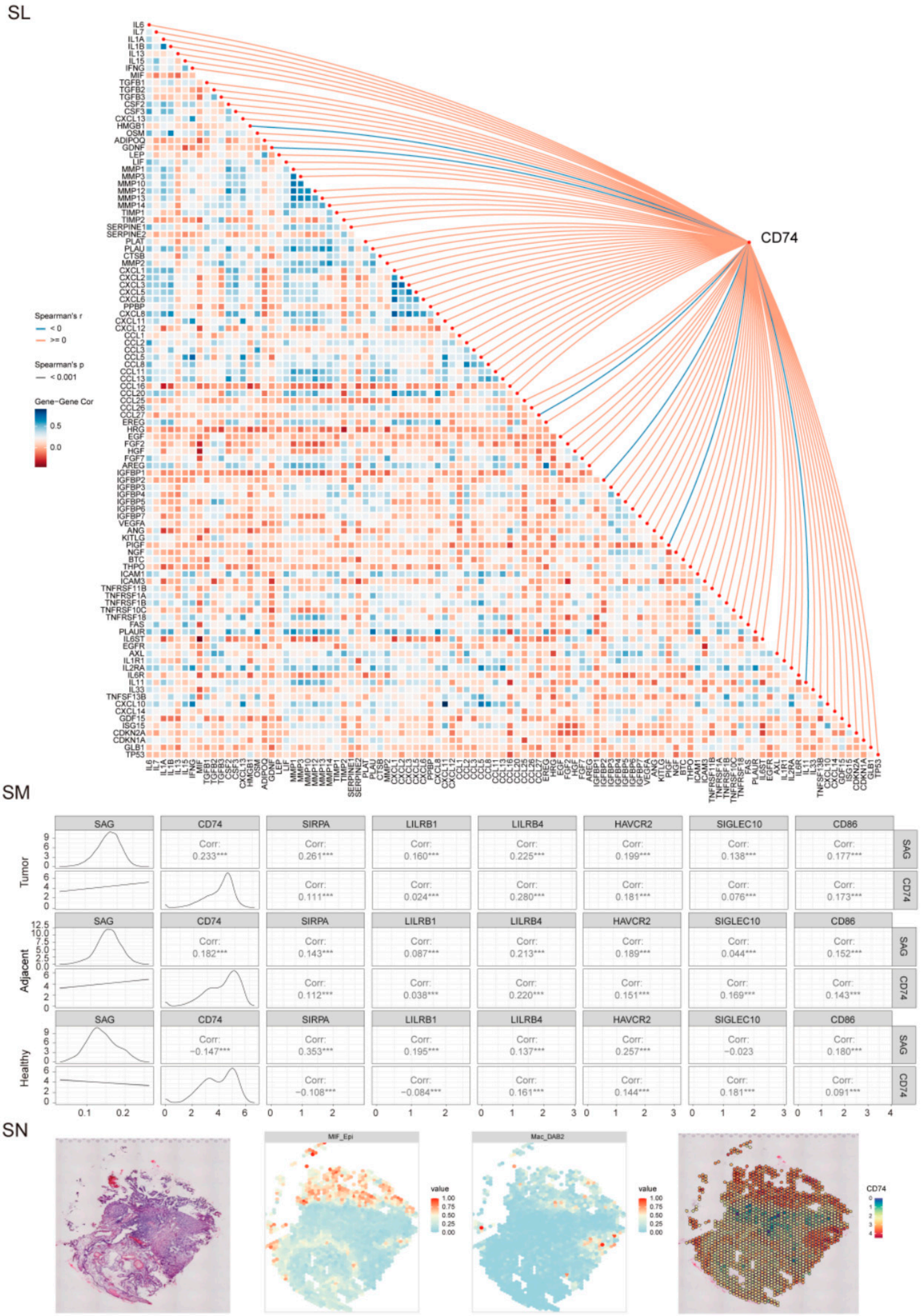

Supplementary Figure S3. Composition and functional heterogeneity of pan-cancer myeloid subpopulations. (**A**) Pie charts showing the composition of pan-cancer myeloid subtypes and their tissue origins. (**B**) Stacked bar plots depicting the relative abundance of myeloid subpopulations across tissue microenvironments (All samples, Tumor, Adjacent, Healthy). (**C–D**) Functional enrichment analyses of 11 myeloid subpopulations in adjacent and healthy control samples. (**E**) Heatmap of metabolic pathway activities across myeloid subpopulations from different tissue origins; colors indicate relative activity levels, and column gaps separate distinct myeloid subsets. (**F**) UMAP projection of pan-cancer DCs

stratified by tissue origin (Tumor, Adjacent, Healthy). (**G**) Bubble plot of canonical marker gene expression across four DC subpopulations. Dot size denotes the proportion of expressing cells, and color intensity reflects relative expression levels. (**H**) Proportional composition of four DC subpopulations stratified by tissue origin (All samples, Tumor, Adjacent, Healthy). (**I**) Comparison of SAG scores among DC subpopulations across Tumor, Adjacent, and Healthy tissues. (**J**) Spearman correlation matrix based on the transcriptomic profiles of four DC subpopulations, with correlation coefficients indicated. (**K**) *CD74* expression across myeloid subpopulations in adjacent and healthy tissues, with subpopulations ranked by their ascending mean expression levels. (**L**) Spearman correlation network of *CD74* with canonical senescence markers and SASP-related genes (orange: positive correlation; blue: negative correlation; line thickness: correlation strength). (**M**) Correlation analyses of SAG scores, *CD74* expression, and canonical myeloid immune checkpoint genes (*SIRPA*, *LILRB1*, *LILRB4*, *HAVCR2*, *SIGLEC10*, *CD86*) across different tissue microenvironments (Tumor, Adjacent, Healthy). (**N**) Representative tissue section displaying H&E staining, proportional distributions of MIF_Epi and Mac_DAB2 subpopulations, and the spatial expression profile of *CD74*. The statistical difference was analyzed by Wilcoxon rank-sum tests with Benjamini-Hochberg adjustment, where **** represents adjusted $P < 0.0001$, *** represents adjusted $P < 0.001$, ** represents adjusted $P < 0.01$, * represents adjusted $P < 0.05$, and ns represents not significant.

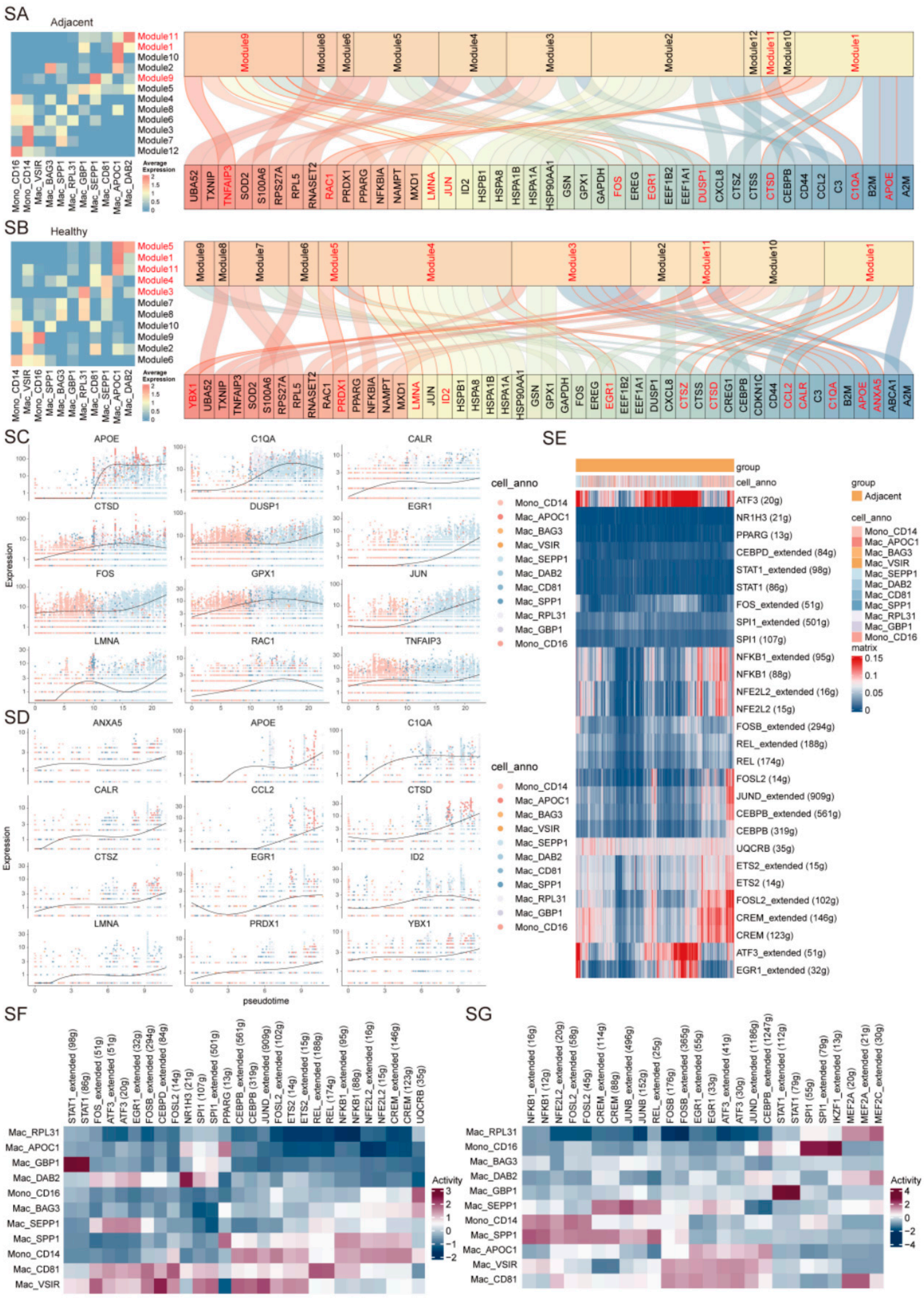

SA
Adjacent
Module11
Module1
Module10
Module2
Module9
Module5
Module4
Module8
Module6
Module3
Module7
Module12
Mono_CD16
Mono_CD14
Mac_VSIR
Mac_BAG3
Mac_SPP1
Mac_RPL31
Mac_GBP1
Mac_SEPP1
Mac_CD81
Mac_APOC1
Mac_DAB2
Average Expression
UBA52
TXNIP
TNFAIP3
SOD2
S100A6
RPS27A
RPL5
RNASET2
RAC1
PRDX1
PPARG
NFKBIA
NAMPT
MXD1
LMNA
JUN
ID2
HSPB1
HSPA8
HSPA1B
HSPA1A
HSP90AA1
GSN
GPX1
GAPDH
FOS
EREG
EGR1
EEF1B2
EEF1A1
DUSP1
CXCL8
CTSZ
CTSS
CTSD
CEBPB
CD44
CCL2
C3
C1QA
B2M
APOE
A2M
SB
Healthy
YBX1
TNFAIP3
CREG1
CDKN1C
CALR
ANXA5
ABCA1
SC
APOE
C1QA
CALR
CTSD
DUSP1
EGR1
FOS
GPX1
JUN
LMNA
RAC1
TNFAIP3
Expression
cell_anno
Mono_CD14
Mac_APOC1
Mac_BAG3
Mac_VSIR
Mac_SEPP1
Mac_DAB2
Mac_CD81
Mac_SPP1
Mac_RPL31
Mac_GBP1
Mono_CD16
SD
ANXA5
APOE
C1QA
CALR
CCL2
CTSD
CTSZ
EGR1
ID2
LMNA
PRDX1
YBX1
pseudotime
SE
group
cell_anno
ATF3 (20g)
NR1H3 (21g)
PPARG (13g)
CEBPD_extended (84g)
STAT1_extended (98g)
STAT1 (86g)
FOS_extended (51g)
SPI1_extended (501g)
SPI1 (107g)
NFKB1_extended (95g)
NFKB1 (88g)
NFE2L2_extended (16g)
NFE2L2 (15g)
FOSB_extended (294g)
REL_extended (188g)
REL (174g)
FOSL2 (14g)
JUND_extended (909g)
CEBPB_extended (561g)
CEBPB (319g)
UQCRB (35g)
ETS2_extended (15g)
ETS2 (14g)
FOSL2_extended (102g)
CREM_extended (146g)
CREM (123g)
ATF3_extended (51g)
EGR1_extended (32g)
group
Adjacent
matrix
SF
STAT1_extended (98g)
STAT1 (86g)
FOS_extended (51g)
ATF3_extended (51g)
ATF3 (20g)
EGR1_extended (32g)
FOSB_extended (294g)
CEBPD_extended (84g)
FOSL2 (14g)
NR1H3 (21g)
SPI1 (107g)
SPI1_extended (501g)
PPARG (13g)
CEBPB_extended (561g)
CEBPB (319g)
JUND_extended (909g)
FOSL2_extended (102g)
ETS2 (14g)
ETS2_extended (15g)
REL_extended (188g)
REL (174g)
NFKB1_extended (95g)
NFKB1 (88g)
NFE2L2_extended (16g)
NFE2L2 (15g)
CREM_extended (146g)
CREM (123g)
UQCRB (35g)
Activity
SG
NFKB1_extended (16g)
NFKB1 (12g)
NFE2L2_extended (20g)
FOSL2_extended (58g)
FOSL2 (45g)
CREM_extended (114g)
CREM (88g)
JUNB_extended (496g)
JUNB (152g)
REL_extended (25g)
FOSB (176g)
FOSB_extended (365g)
EGR1_extended (55g)
EGR1 (33g)
ATF3_extended (41g)
ATF3 (30g)
JUND_extended (1186g)
CEBPB_extended (1247g)
STAT1_extended (112g)
STAT1 (79g)
SPI1 (55g)
SPI1_extended (79g)
IKZF1_extended (13g)
MEF2A (20g)
MEF2A_extended (21g)
MEF2C_extended (30g)
Activity

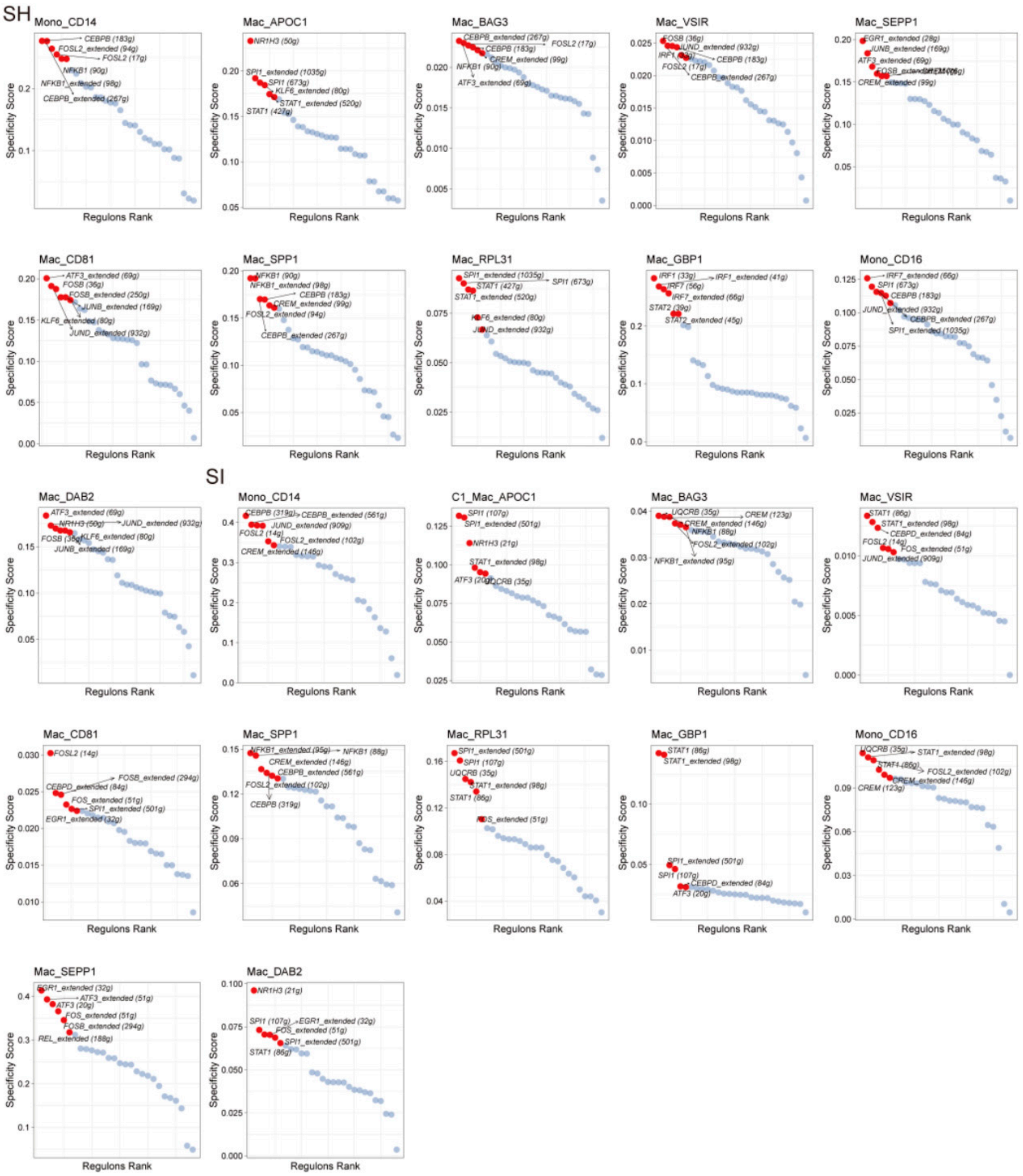
SH
Mono_CD14
Mac_APOC1
Mac_BAG3
Mac_VSIR
Mac_SEPP1
Mac_CD81
Mac_SPP1
Mac_RPL31
Mac_GBP1
Mono_CD16
Mac_DAB2
SI
Mono_CD14
C1_Mac_APOC1
Mac_BAG3
Mac_VSIR
Mac_CD81
Mac_SPP1
Mac_RPL31
Mac_GBP1
Mono_CD16
Mac_SEPP1
Mac_DAB2
Specificity Score
Regulons Rank

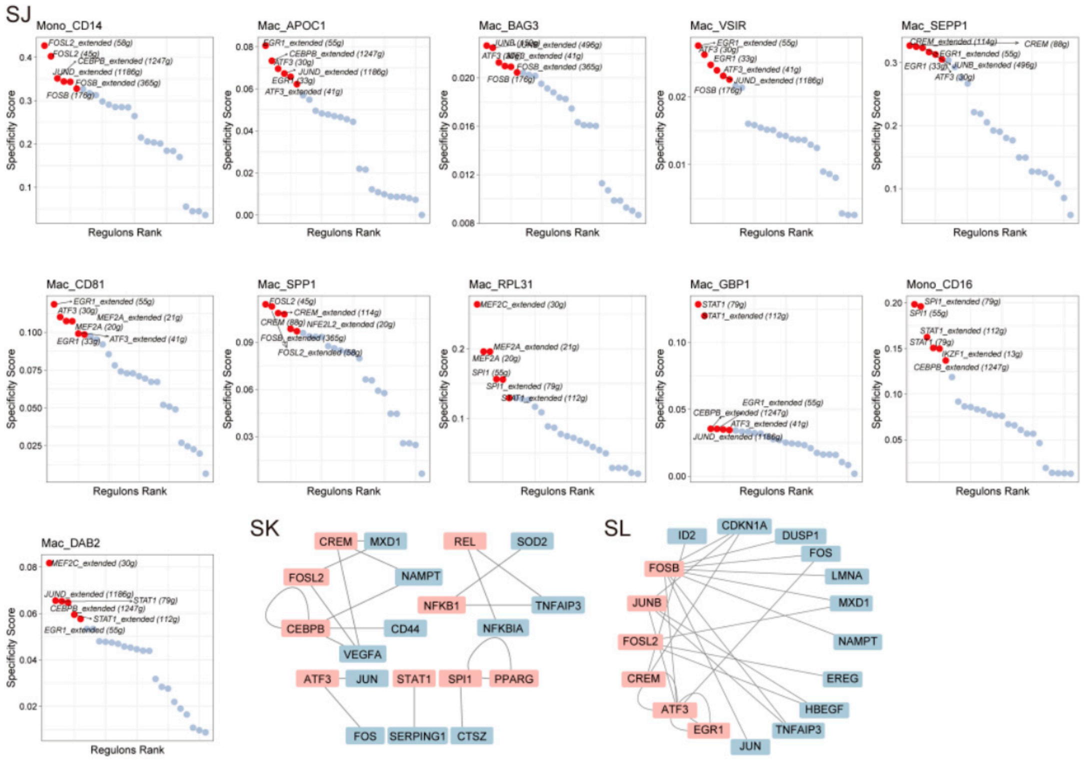

Supplementary Figure S4. Senescence-associated transcriptional modules and regulatory networks of Mono/Macro cells across tissue microenvironments. (**A–B**) In adjacent (A) and healthy (B) tissues, heatmaps (left) show the average expression of gene modules across Mono/Macro subpopulations (color denotes expression intensity), while Sankey diagrams (right) illustrate the distribution of key senescence genes across modules. (**C–D**) In adjacent (C) and healthy (D) tissues, dynamic expression changes of representative senescence key genes within modules upregulated in terminally differentiated Mono/Macro subpopulations along pseudotime. (**E**) Heatmap of inferred regulon activities in Mono/Macro subpopulations from adjacent tissues. Columns are annotated by tissue origin and cell subpopulation; rows represent differentially active regulons, and the color scale indicates normalized regulon activity. (**F–G**) Regulon activity levels across Mono/Macro subpopulations in adjacent (F) and healthy (G) tissues. (**H–J**) Regulon ranking of myeloid subpopulations in tumor (H), adjacent (I), and healthy (J) tissues. The top six key drivers with the highest specificity scores are highlighted in red; the y-axis indicates specificity scores. (**K–L**) Transcriptional regulatory networks linking core transcription factors and senescence key genes in adjacent (K) and healthy (L) tissues. Red nodes denote core transcription factors, and blue nodes represent downstream senescence-associated target genes.

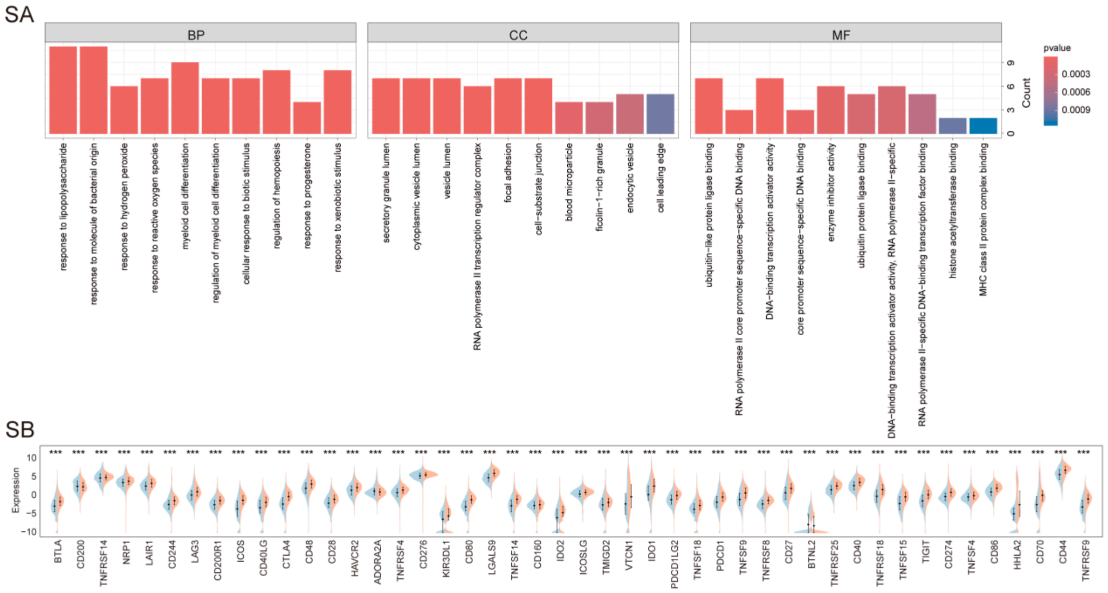

Supplementary Figure S5. Functional annotation and immune checkpoint characteristics associated with MSAG. (**A**) Bar plots showing significantly enriched terms for the 35 constituent genes of MSAG across biological process (BP), cellular component (CC), and molecular function (MF) categories. (**B**) Differential expression of immune checkpoint genes between the two subtypes. The statistical difference was analyzed by Wilcoxon rank-sum tests, where *** represents $P < 0.001$, ** represents $P < 0.01$, * represents $P < 0.05$, and ns represents not significant.

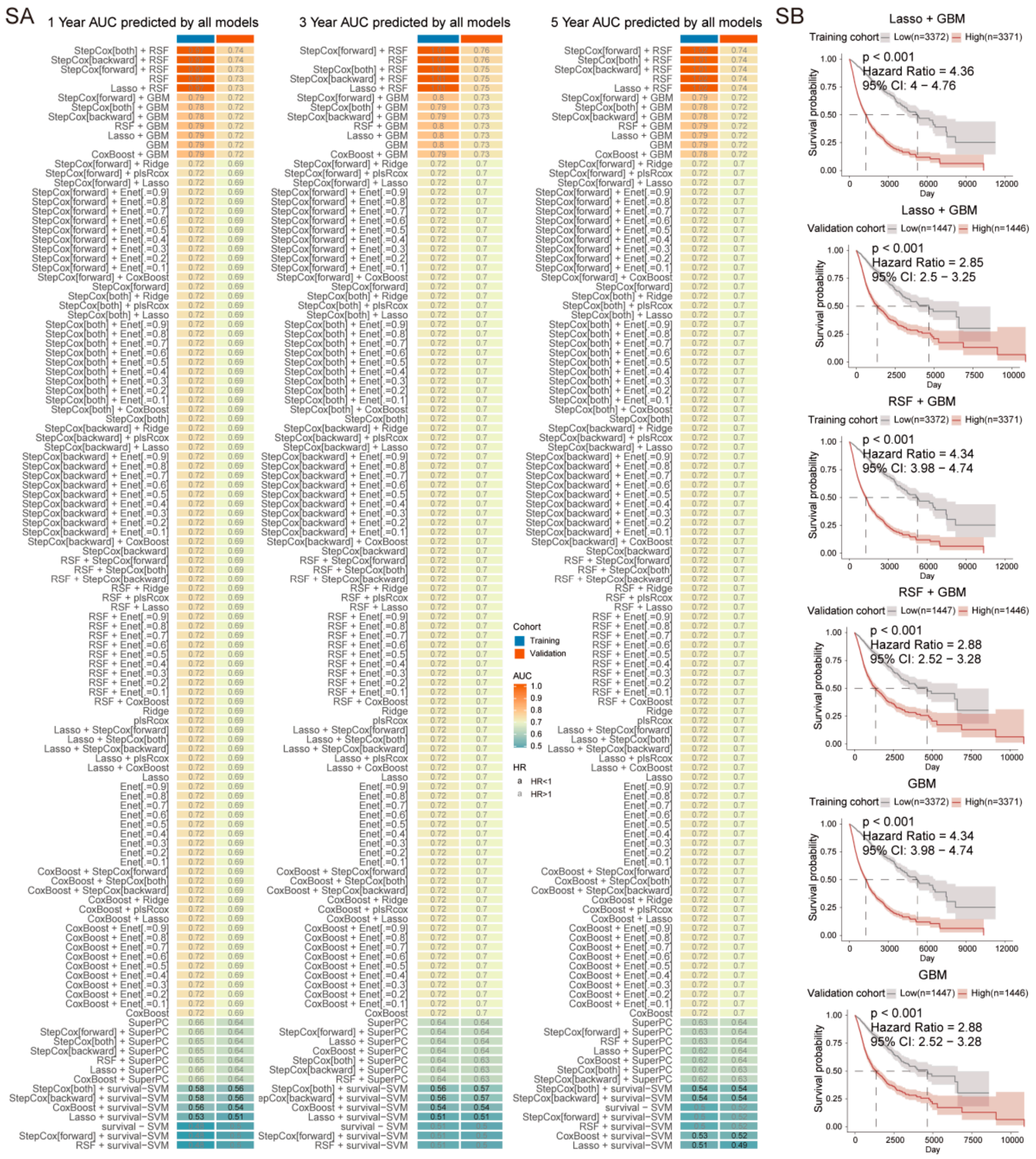

Supplementary Figure S6. Performance evaluation of the *MSAG.SIG* prognostic models. (**A**) Time-dependent AUC values at 1, 3, and 5 years for 117 machine-learning combination models in the TCGA training and internal validation cohorts. (**B**) Kaplan-Meier survival analyses of candidate GBM-based combination models in the training and internal validation cohorts.

Supplementary Table 1. Analyzed cell types and marker gene sets. This table lists the evaluated cell categories and their corresponding marker genes used for enrichment score normalization.

Supplementary Table 2. Detailed sources of senescence-associated genes. This table details the SAGs compiled from public databases and published literature.

Supplementary Table 3. Candidate prognostic genes. This table provides the genes identified via univariate Cox regression from the TCGA dataset, which were used for subsequent machine learning model construction.

Supplementary Table 4. Consensus senescence-associated genes. This table lists the final curated set of 769 senescence genes, each supported by at least two independent databases or literature sources.

Supplementary Table 5. Complete list of CellChat-derived key interaction genes, including ligand and receptor genes involved in significantly enriched intercellular communication pathways ($P < 0.05$), used for overlap analysis with SAGs.
